# How to Implement a Ketogenic Diet Therapy in Developing Countries: Colombian Consensus on Ketogenic Diet Therapy Use in the Management of Adults with Drug-Resistant Epilepsy

**DOI:** 10.3390/nu18203458

**Published:** 2026-10-21

**Authors:** Jean Paul Vergara-Aguilar, Angélica Lizcano, Adriana Martinez-Perez, Yamile Calle-López, Mackenzie C. Cervenka, Carlos A. Clavijo-Prado, Juan Camilo Cotuá-Urzola, Walter González, Dahyana K. González-Velásquez, Malena C. P. Grillo-Ardila, Paula Martinez-Micolta, Lina M. Ramírez, Nataly Rincón-Franco, Juliana Vargas Osorio, Lyda V. Villamil-Osorio, Adriana Tanner

**Affiliations:** 1 Department of Neurology, Fundación Hospital Infantil Universitario de San José, Bogotá 111221, Colombia; 2 Neurology Program, Fundación Universitaria de Ciencias de la Salud (FUCS), Bogotá 111411, Colombia; 3 Epilepsy Surgery Program, Neurocentro, Calle 12 #18-24 Torre 3 Piso 3, Pereira 660003, Colombia; 4 Epilepsy Program, CONEURO, Cúcuta 540003, Colombia; 5 Department of Neurology, Clínica FOSCAL-Fundación Oftalmológica-Centro Médico Carlos Ardila Lulle, Floridablanca 681004, Colombia; 6 Faculty of Medicine, Universidad de Antioquia UDA, Calle 70 No. 52-21, Medellín 050010, Colombia; 7 Neurology Section, Hospital Universitario San Vicente Fundación, Medellín 050010, Colombia; 8 Department of Neurology, Johns Hopkins University School of Medicine, Baltimore, MD 21205, USA; 9 Epilepsy Program, Instituto Neurológico del Pacífico, Cali 760043, Colombia; 10 Neurology Section, Faculty of Medicine, Universidad del Valle, Cali 760032, Colombia; 11 Neurophysiology Unit, Neurocardiología del Caribe, Sincelejo 700001, Colombia; 12 Epilepsy and Epilepsy Surgery Service, Clínica Reina Sofía Colsanitas, Bogotá 110121, Colombia; 13 Faculty of Basic Sciences, Fundación Universitaria de Ciencias de la Salud (FUCS), Bogotá 111411, Colombia; 14 Neurophysiology Unit, Clínica Medilaser, Neiva 410010, Colombia; 15 Neurophysiology Unit, Clínica Uros, Neiva 410010, Colombia; 16 Functional Neurology Section, Clínica CLAM, Bogotá 111111, Colombia; 17 Department of Neurology, Hospital Universitario Fundación Santa Fe de Bogotá, Bogotá 110111, Colombia; 18 Faculty of Exact and Natural Health Sciences, Universidad Libre de Colombia, Pereira 660003, Colombia; 19 Fundación Fundem, Bogotá 110121, Colombia; 20 Clinical Nutrition, Asociación Médica de Cuidados Especiales (AMECE), Medellín 050021, Colombia; 21 Clinical Nutrition, Universidad Corporación en Estudio de la Salud (CES), Medellín 050021, Colombia; 22 Neurology Service, Hospital Militar Central, Bogotá 110231, Colombia; 23 Neurology and Neurophysiology Service, Mioneuro S.A.S., Ibagué 730006, Colombia; 24 Neurophysiology Service, Sociedad Médico Quirúrgica del Tolima, Ibagué 730006, Colombia; 25 Neurophysiology and Epilepsy, A.S. Neurophysiology Services, PLLC, Grand Rapids, MI 49525, USA

**Keywords:** drug-resistant epilepsy, ketogenic diet therapy, adults, super-refractory status epilepticus, dietary therapies

## Abstract

Background/Objectives: Drug-resistant epilepsy affects up to one-third of patients with epilepsy and is associated with higher morbidity, mortality, trauma events, psychosocial challenges, and decline in quality of life. In Latin America, disease burden is high and access to advanced therapies remains limited. Ketogenic Diet Therapy (KDT) has been shown to be beneficial in adults, but its implementation requires standardized protocols and experienced multidisciplinary teams. This study aimed to develop practical, evidence-based recommendations to guide the indication, implementation, and follow-up of KDT in adults with drug-resistant epilepsy through expert consensus in Colombia. Methods: A modified version of the Nominal Group Technique was used to develop, discuss, and vote on management concepts. Clinical questions were structured using the PICO framework. Literature searches were conducted in PubMed and EMBASE for articles published since 2015, and the evidence was integrated with the experience of a multidisciplinary expert panel. Results: Twelve recommendations were issued, covering commercial formulas, induction and follow-up protocols, individualized dietary interventions, safety, diagnostic support, nutritional supplementation, and drug-diet interactions. The consensus highlights the need for interdisciplinary teams, structured education, early adherence strategies, and close follow-up, and the role of enteral emergency formulas in intensive care settings. Conclusions: Evidence around KDT shows it can be implemented safely as an adjunctive treatment for adults with drug-resistant epilepsy and is reasonable to consider following the failure of two adequate antiseizure medications. Structured implementation may reduce seizure frequency, improve quality of life, and reduce the pharmacological burden. Multicenter prospective studies are needed to further assess efficacy, safety, and resource utilization. These recommendations contribute to closing the gap between evidence and clinical practice.

## 1. Introduction

The International League Against Epilepsy (ILAE) defines refractory or drug-resistant epilepsy (DRE) as the failure of adequate trials of two tolerated, appropriately chosen and used antiseizure drug schedules, whether as monotherapies or in combination, to achieve sustained seizure freedom [1].

DRE affects approximately one-quarter to one-third of patients with epilepsy and is associated with an increased risk of premature mortality, traumatic injuries, psychosocial disruption, and significant deterioration in quality of life [2]. Although individuals with DRE may temporarily enter remission, seizure recurrence remains frequent. Therefore, early identification of DRE and timely referral to specialized epilepsy centers are essential to evaluate therapeutic strategies beyond further pharmacological escalation [3,4]. After failure of two appropriately selected antiseizure medications, the probability of achieving seizure freedom with additional pharmacological trials is significantly reduced [3]. Accordingly, recent clinical guidelines emphasize early referral to tertiary or comprehensive epilepsy centers after failure of two antiseizure medications, allowing diagnostic reassessment, optimization and adjustment of antiseizure medications, and evaluation of epilepsy surgery, neuromodulation—including vagus nerve stimulation (VNS), deep brain stimulation (DBS), and responsive neurostimulation (RNS)—and ketogenic diet therapy (KDT) as part of a comprehensive, structured, multidisciplinary management strategy [3,4,5,6,7,8]. In this context, clinicians require therapeutic strategies that extend beyond sequential antiseizure medication trials.

The positioning of ketogenic diet therapy within current international recommendations and its relationship to epilepsy surgery and neuromodulation are summarized in Supplementary Table S1.

In adults with DRE, KDT represents a safe and effective non-pharmacological therapeutic option when other interventions are not feasible, unavailable, or unsuccessful [5,9,10]. Its implementation requires structured protocols and multidisciplinary teams that include neurologists or epileptologists and nutritionists trained in ketogenic therapies [5].

Less restrictive dietary variants, such as the Modified Atkins Diet (MAD) and the Low Glycemic Index Treatment (LGIT), may improve adherence in adults while maintaining clinically meaningful efficacy. Adherence is a major determinant of treatment success and may be limited by adverse effects, dietary restrictiveness, meal-preparation burden, lack of motivation, and cost; structured education, monitoring, and individualized adjustments are therefore essential [5,10,11].

S1.11. Summary Flow Chart for modified NGT method used [1,2,3].

Within this framework, classical and modified ketogenic therapies are increasingly considered earlier in the treatment algorithm because of their potential to reduce seizure frequency, decrease antiseizure medication burden, and contribute to improved quality of life [5,12,13].

Current clinical evidence supports the use of ketogenic therapies in adults with DRE, including randomized clinical trials, systematic reviews, and international consensus recommendations [5,12,13]. The randomized controlled trial by Zare et al. showed that adjunctive treatment with the Modified Atkins Diet reduced seizure frequency compared with the control group in adults with refractory epilepsy [13]. Systematic reviews, including Cochrane analyses, confirm that ketogenic therapies reduce seizure frequency, although most of the available data derive from pediatric populations [12]. International consensus recommendations for adults further support their safety and potential effectiveness when administered by trained multidisciplinary teams using structured protocols [5].

Additional benefits reported in adult populations include improvement in quality of life [13], usefulness in the management of super-refractory status epilepticus (SRSE) [14,15,16,17], and the possibility of reducing antiseizure medication burden [18]. Overall, adherence and safety profiles are acceptable when treatment is implemented with appropriate monitoring and less restrictive dietary variants such as MAD and LGIT [5,10,19]. Adverse events such as dyslipidemia, gastrointestinal symptoms, metabolic acidosis, nephrolithiasis, and micronutrient deficiencies are generally manageable with structured monitoring.

Potential barriers to implementation include adverse effects, treatment complexity, adherence challenges, interactions with specific medications such as propofol and carbonic anhydrase inhibitors, carbohydrate-containing excipients, and limited training of healthcare teams in ketogenic diet therapy protocols [15].

Commercial and enteral ketogenic formulas facilitate the precise induction of ketosis, improve macronutrient consistency, and enhance adherence and tolerability, particularly in intensive care settings or when oral administration is not feasible [15].

Although ketogenic diet therapy has historically been used in epilepsy management, its implementation in adults remains limited in routine clinical practice. Optimizing its use requires trained multidisciplinary teams, standardized protocols, structured monitoring, and guidance adapted to available healthcare resources [5,10].

International consensus statements provide recommendations on candidate selection, treatment initiation, monitoring, supplementation, adverse-event management, and discontinuation of ketogenic diet therapy in adults [5,9]. However, their implementation may be challenging in healthcare systems with heterogeneous access not only to epilepsy surgery and neuromodulation, but also to trained ketogenic diet teams, laboratory monitoring, commercial ketogenic products, and standardized pharmacy support [10,15,20]. In Colombia, additional practical barriers may include socioeconomic constraints affecting food selection, difficulties maintaining long-term adherence, and variability in the organization of hospital-based and outpatient ketogenic diet therapy services.

This Colombian consensus was developed to complement existing international recommendations by providing practical, evidence-informed guidance for the selection, initiation, implementation, and follow-up of ketogenic diet therapy in adults with DRE. It offers an adapted, implementation-oriented guideline for adult ketogenic diet therapy within a middle-income Latin American healthcare model. Thus, these recommendations add to the current indexed literature by providing practical advice on treatment operations for adults with DRE in resource-variable settings and may serve as a benchmark for other Latin American countries facing similar healthcare organization and access-related challenges.

## 2. Materials and Methods

### 2.1. Expert Panel and Consensus Design

Fourteen Colombian experts participated in the consensus process, including 11 neurologist-epileptologists and three nutritionists. Panel members were selected based on their expertise, academic contributions, research experience, and clinical experience in the management of adults with drug-resistant epilepsy using ketogenic diet therapy.

The consensus was developed through nine phases using a modified Nominal Group Technique (NGT) supported by information and communication technology tools [21]. The core components of the NGT were preserved: independent generation of responses, review of peer contributions with an opportunity to revise the initial responses, structured clarification of concepts and positions, and formal voting [21].

The classical Nominal Group Technique (NGT) is a structured face-to-face consensus method comprising four core stages: silent generation of ideas, round-robin presentation, structured clarification, and individual voting or ranking. In the present consensus, the NGT was modified to allow remote participation and to integrate evidence review into the consensus process. Independent responses and anonymous peer review were conducted asynchronously through a web-based platform, replacing the traditional face-to-face round-robin stage, while structured clarification was conducted by videoconference. Voting was performed iteratively using online tools and predefined agreement thresholds. Thus, the modified approach preserved the essential components of independent response generation, structured clarification, and formal voting while adapting their delivery to a nine-phase, evidence-informed, hybrid asynchronous–synchronous process [22,23].

**Figure 1:**
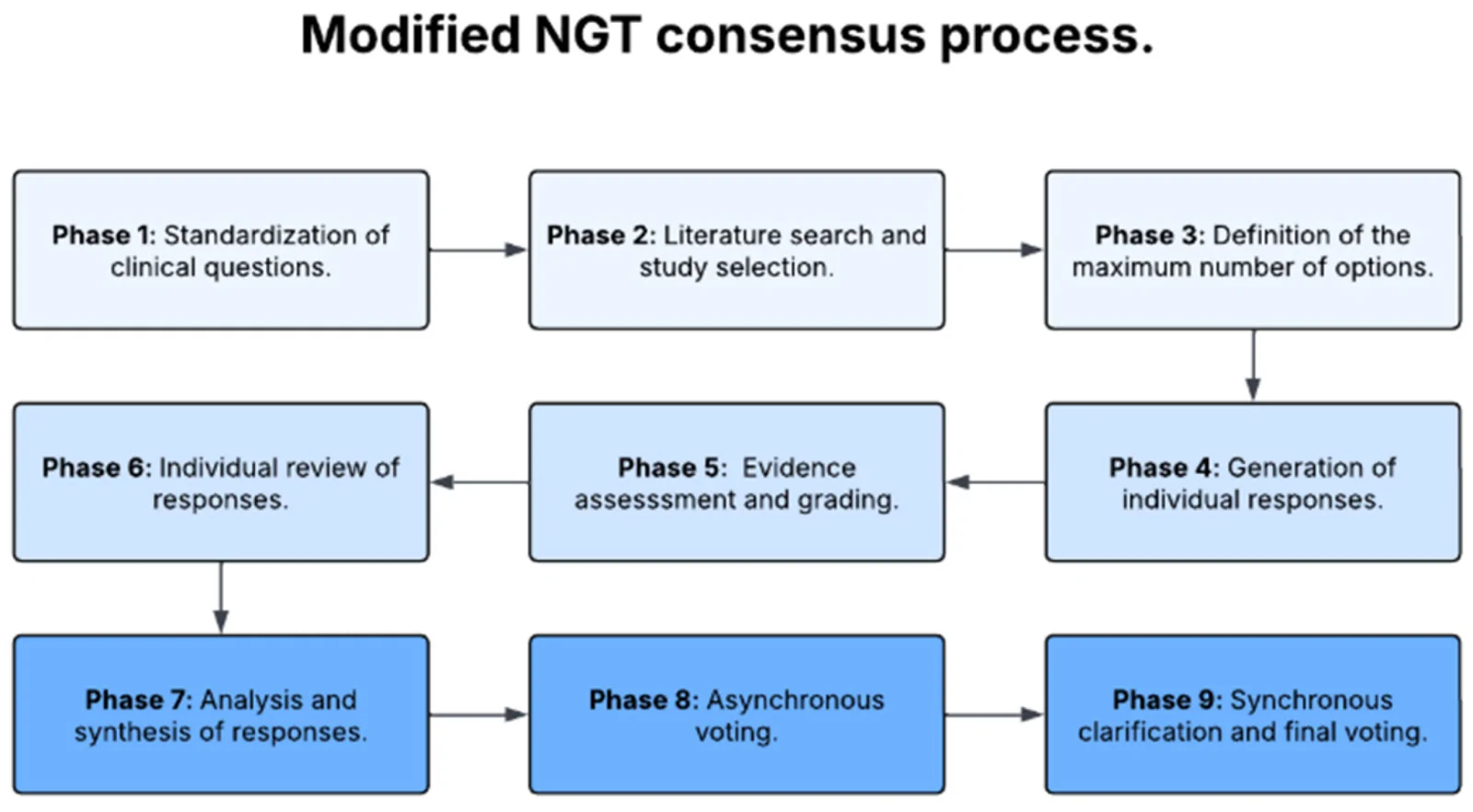
Summary Flow Chart for modified NGT method.

A detailed description of the nine phases of the modified Nominal Group Technique consensus process is provided in Supplementary Methods S1 and a flow diagram of the modified NGT process is presented in Figure 1.

### 2.2. Clinical Questions and Evidence Review

The clinical questions were structured according to the PICO format. Search strategies were developed for each question, and literature searches were conducted in PubMed and EMBASE. The complete search strategies for each clinical question and database are provided in Supplementary S2 Complete Search Strategies for Each Clinical Question and Database.

Although 13 clinical questions were initially formulated to address clinically relevant aspects of ketogenic diet therapy in adults with drug-resistant epilepsy, Questions 12 and 13 were ultimately integrated into a single recommendation in the final manuscript. These questions addressed the laboratory parameters to be monitored and the recommended frequency of monitoring, respectively. Because both components are closely interrelated and constitute a single clinical decision-making process in routine practice, they were considered together in the final recommendation.

Articles published from 2015 onward were considered because most of the evidence specifically addressing ketogenic diet therapy in adults is recent, whereas earlier evidence had been evaluated in previous systematic reviews, including Cochrane reviews. During manuscript revision, selected references were updated through June 2026 to incorporate more recent evidence; the original database searches were not rerun. Eligible study designs included systematic reviews, randomized clinical trials, non-randomized trials, cohort studies, case–control studies, and case series.

For each question, the searches were conducted by two independent reviewers, and the resulting strategies were consolidated for each database. The eligibility of the identified records was assessed manually. Disagreements were resolved by consensus and, when necessary, through consultation with a third reviewer with postgraduate training in clinical epidemiology and experience in evidence appraisal and scientific research.

The methodological quality and risk of bias of the included evidence were assessed according to study design. Clinical practice guidelines were evaluated using the AGREE II instrument [24]; systematic reviews using ROBIS [25]; randomized controlled trials using the Cochrane Risk of Bias 2 tool [26]; non-randomized studies of exposures using ROBINS-E [27]; and cohort and case–control studies using the Newcastle–Ottawa Scale [28]. Methodological quality and risk-of-bias assessments were performed independently by two reviewers, and disagreements were resolved by consensus.

Articles published from 2015 onward were considered because most of the evidence specifically addressing ketogenic diet therapy in adults is recent, whereas earlier evidence had been evaluated in previous systematic reviews, including Cochrane reviews. During manuscript revision, selected references were updated through June 2026 to incorporate more recent evidence; the original database searches were not rerun.

A detailed description of the consensus methodology—including expert panel selection, the modified Nominal Group Technique framework, standardization of the clinical questions, literature search and study selection, evidence appraisal and grading, generation and synthesis of individual responses, and the asynchronous and synchronous voting procedures—is provided in Supplementary Methods S1.

### 2.3. Development and Grading of the Recommendations

For each clinical question, experts were required to select one option as the proposed recommendation. Each participant received the clinical questions, instructions for submitting responses through the web-based platform, and the full text of the articles selected during the evidence review. Participants could also submit additional references considered relevant to the question.

The class of recommendation and level of evidence were assigned using the framework of the American College of Cardiology Foundation/American Heart Association Task Force on Practice Guidelines [29]. This framework was selected because it allows the strength of the recommendation and the supporting level of evidence to be assessed separately, which is particularly relevant in clinical scenarios supported by heterogeneous evidence.

After submitting their initial responses, participants reviewed the responses proposed by the other experts and could revise their own answers. The final responses were reviewed and synthesized using affinity matrices. Responses that did not comply with the required structure of a recommendation were excluded, and responses with similar content, wording, and organization were consolidated into individual recommendation options.

### 2.4. Voting and Consensus Rules

During Phase 8, the experts completed an asynchronous voting process through the web-based platform.

During Phase 9, the panel participated in two synchronous videoconference meetings. The recommendation options were discussed, questions regarding their wording were addressed, and voting was conducted on the recommendation statements, their class of recommendation, and their level of evidence.

A maximum of three voting rounds was allowed. An option was approved when it reached at least 80% agreement. Options receiving 30% agreement or less were excluded from the subsequent voting round. If the predefined agreement threshold was not reached after the third round, the option receiving the highest number of votes was retained as the final option.

When agreement was not reached during the first round, or when two or more options received similar vote counts, a structured clarification process was initiated. Participants could present arguments in favor of or against each option before the subsequent voting round.

For each clinical question, the proposed recommendation statement was voted on first. The class of recommendation was subsequently submitted to voting using the American College of Cardiology Foundation/American Heart Association framework [30,31]. After the class of recommendation reached the predefined agreement threshold of at least 80%, the wording was reviewed and amended, when necessary, to ensure consistency with the approved class. Finally, the level of evidence was voted on using the final approved wording of the recommendation.

## 3. Results—Recommendations

The consensus process addressed 12 clinical questions and generated 12 recommendations. All recommendations reached the predefined consensus threshold of at least 80% agreement among panel members. Of the 12 recommendations, 11 were classified as Class I and one as Class IIa; two were supported by Level B-R evidence and 10 by Level B-NR evidence.

**Question 1: *In adult patients with drug-resistant epilepsy who want to achieve a clinically significant reduction in seizures, which are the relevant clinical indications to prescribe ketogenic diet therapy with a commercial formula?***

**Recommendation 1**

It is reasonable to use ketogenic diet therapy with a commercial formula in adult patients with drug-resistant epilepsy in the following scenarios:

- Clinical requirements of rapid and sustained reduction in ketosis (high ketogenic ratio), including refractory or super-refractory status epilepticus (SRSE) [32], resulting from new-onset refractory status epilepticus (NORSE) [33] including febrile infection related epileptic syndrome (FIRES) [34], or other seizure types and epilepsy syndromes, or in patients with an absolute need to maintain an accurate control of calorie intake and macronutrients to accomplish an effective therapeutic response.
- Difficulties adhering to ketogenic diet therapy in its different variants, with constipation, poor tolerance or the need for an alternate feeding route in order to optimize adherence, tolerance and treatment efficacy.
- Difficulties in selecting the most appropriate ketogenic diet modality, after exclusion of underlying metabolic disorders that may contraindicate ketogenic diet therapy.

**Class IIa; Level B-R**

**Question 2: *In adult patients with SRSE or cluster seizures, in whom ketogenic diet is used to control seizures, what are the indications to prescribe ketogenic diet with commercial formula?***

**Recommendation 2**

In adult patients with SRSE or cluster seizures, the recommendation is to prescribe a ketogenic diet with commercial formula in the following cases:

- The SRSE diagnosis has been confirmed and there is no resolution following the sequential use of benzodiazepines, second-line antiseizure medications, and infused anesthetic agents; moreover, there are no absolute clinical or metabolic contraindications.
- In cluster seizures that have been refractory to conventional pharmacotherapy, there is a high risk of progression to status epilepticus and the same clinical, nutritional and logistic conditions required for managing SRSE are necessary.
- The patient presents with hemodynamic stability, preserved kidney and liver function, ketosis and electrolyte monitoring available, no use of propofol over the past 24 h, and availability of a skilled clinical multidisciplinary team.
- There is an urgent need to achieve effective ketosis via elevated ketogenic ratios (≥2:1), with accurate calorie intake and macronutrient control, using formulas adapted to the patient’s clinical condition.
- The oral administration of the classical ketogenic diet is impossible, hence requiring enteral nutrition or an accurate and standardized liquid formulation, in the intensive care unit (ICU).

**Class I; Level B-R**

**Note:** A practical treatment algorithm for the initiation and management of ketogenic diet therapy in adults with super-refractory status epilepticus is provided in Appendix A.

**Question 3: *In adult patients with drug-resistant epilepsy, who are candidates for ketogenic diet therapy, which elements should be included in the initial protocol for ketogenic diet therapy, which elements should be included to achieve an adequate level of adherence?***

**Recommendation 3**

In adult patients with drug-resistant epilepsy, who are candidates for ketogenic diet, a protocol including the following considerations is recommended to maximize ketogenic treatment adherence:

- Specialized neurology assessment, comprehensive nutritional assessment (including anthropometrics, biochemical and lifestyle tests), exclusion of clinical or metabolic contraindications and identification of risk factors for poor adherence.
- Education sessions for the patient and caregivers before the initiation of treatment, focusing on treatment benefits, risks, dietary options and practical training in label reading, in addition to close support during the first few weeks by a multidisciplinary team.
- Recipe books, shopping lists, food and seizures journals, everyday menu examples (work, study, travel), as well as meal plans adapted to the patient’s environment and lifestyle.
- Possibility to choose among different types of diet (classical, medium chain triglyceride-based—MCT—, modified Atkins), with gradual ketogenic ratio adjustments according to tolerance and response, and explicit criteria to switch between dietary modalities when adherence barriers are identified.
- Structured follow-up through medical, nutritional and psychological visits, control of ketones, lipids and liver/kidney parameters, and individualized dietary adjustments based on objective data (seizure journal, ketonuria, adverse effects).

**Class I; Level B-NR**

***Note*: Check Supplementary GPI S2:** Absolute and relative contraindications for starting ketogenic diet therapy

**Question 4**

***In adult hospitalized patients*** *(**emergency department, inpatient floor or ICU**) **with drug-resistant epilepsy receiving ketogenic diet therapy, which co-administered medications are considered at high risk of causing serious interactions or may prevent stable ketosis** (**β-hydroxybutyrate > 2 mmol/L**)**?***

**Recommendation 4**

In **adult hospitalized patients** (emergency department, inpatient floor or ICU) with drug-resistant epilepsy, receiving ketogenic diet therapy, the recommendation is to assess the medications and intravenous (IV) solutions administered to prevent drug interactions affecting ketosis or representing severe risks, for instance:

- Propofol, which directly inhibits fatty acid oxidation and mitochondrial function, which could precipitate propofol infusion syndrome and therefore should be discontinued at least 12–24 h before starting the diet.
- Corticosteroids, immunosuppressors or medications that cause insulin resistance and gluconeogenesis due to inhibition of ketone bodies production and metabolic dysfunction.
- Syrups, oral suspensions, IV glucose solutions, sugar-containing antibiotics, hypercaloric enteral formulas and excipients containing glucose, sorbitol, lactose, mannitol or alcohols, which interfere directly with ketogenesis.
- Carbonic anhydrase inhibitors (such as topiramate and zonisamide), due to their association with metabolic acidosis, nephrolithiasis and increased risk of adverse events when combined with ketogenic diet therapy.
- Valproate, phenobarbital, lamotrigine, clobazam, phenytoin and carbamazepine, which may be reduced or potentiated when starting the diet, requiring dose adjustments and close pharmacological monitoring.

**Class I; Level B-NR**

**Note:** Additional practical recommendations for the hospitalized implementation of ketogenic diet therapy are provided in GPI 5.

**Question 5: *In adult ambulatory patients with drug-resistant epilepsy receiving ketogenic diet therapy, which medications commonly used in the outpatient setting—if co-administered—are considered high risk for the development of severe interactions or may prevent stable ketosis (β-hydroxybutyrate > 2 mmol/L)?***

**Recommendation 5**

In **adult ambulatory patients** with drug-resistant epilepsy, receiving ketogenic diet therapy, the recommendation is to carefully review all the medications co-administered in the outpatient setting to identify those that may interfere with ketosis or increase the risk of adverse events, such as:

- Oral or parenteral steroids that inhibit ketosis by inducing insulin resistance and activating gluconeogenesis.
- Syrups, suspensions, and solutions containing excipients such as glucose, fructose, sorbitol, lactose, glycerol or maltodextrin, including anti-cough syrups, suspension antibiotics and liquid multivitamin preparations.

**Class I; Level B-NR**

Note: Additional practical recommendations for the outpatient implementation of ketogenic diet therapy, including pretreatment assessment, dietary initiation, prevention of complications, monitoring, and treatment progression, are provided in Appendix A. Algorithm for the Initiation of Ketogenic Diet Therapy in Adults in the Outpatient Setting. Good Practice Item S3. Recommendations for the Implementation of Ketogenic Diet Therapy in the Outpatient

**Question 6: *In adult patients with drug-resistant epilepsy receiving ketogenic diet therapy, which are the minimum requirements for healthcare providers that should comprise the structured follow-up team for improved treatment adherence, epileptic seizures control, and for managing side effects?***

**Recommendation 6**

In adult patients with drug-resistant epilepsy, receiving ketogenic diet therapy, the recommendation is to have available a team of specialized healthcare professionals to optimize the results of the ketogenic diet therapy. As a minimum, this team shall include:

- Nutritionists specially trained in ketogenic diet therapy, responsible for designing, adjusting and monitoring the nutritional plan, controlling the ketogenic ratio, preventing nutritional deficiencies and educating the patient and family.
- Neurologist or epileptologist, responsible for adjusting the pharmacological therapy, discussing the clinical objectives of the diet, assessing the clinical response and managing any epilepsy and KDT-associated adverse events.
- Nursing staff trained in ketogenic diet therapy, in charge of coordinating the administration of the diet and medications, providing continuous education, monitoring any adverse events and maintaining frequent communication with patients and families.
- Psychologists or psychiatrists to address emotional, behavioral or mental health issues that may impact treatment adherence and continuity in an ambulatory setting.
- Social worker or clinical manager to assist in overcoming any socioeconomic barriers, coordinating the delivery of supplies and facilitating continuity of treatment in vulnerable populations.
- Additional specialists such as internist (to manage any comorbidities), gastroenterologist (for gastrointestinal issues) or speech therapist (in case of swallowing disorders), in accordance with the patient’s needs.

**Class I; Level B-NR**

**Question 7**

***In adult patients with drug-resistant epilepsy for whom the classical ketogenic diet therapy is not the best option, which of the modified ketogenic therapies*** *(**modified Atkins, medium chain and low glycemic index treatment**) **is the most advisable?***

**Recommendation 7**

In adult patients with drug-resistant epilepsy for whom the classical ketogenic diet therapy is not the best option, the recommendation is to individualize the choice of modified ketogenic diet (MAD, MCT or LGIT), based on the patient’s clinical profile, their medical comorbidities, social context, expected adherence, tolerance and the experience of the treating physician.

**Class I; Level B-NR**

**Note:** The main ketogenic diet therapy modalities used in adults with epilepsy, including their characteristics, potential indications, tolerability, and implementation considerations, are summarized in Supplementary Table S3 Types of ketogenic diet therapy for patients with epilepsy

**Question 8: *In patients with drug-resistant epilepsy and high cardiovascular risk who receive ketogenic diet therapy, which should be the most appropriate composition of the fats administered to favor the reduction in LDL cholesterol?***

**Recommendation 8**

In patients with drug-resistant epilepsy and high cardiovascular risk who receive ketogenic diet therapy, the recommendation is to adapt ketogenic diet therapy to a Mediterranean-style dietary pattern, prioritizing monounsaturated fats (such as olive oil, avocado, and nuts), omega-3 polyunsaturated fats (such as oily fish, flaxseed, and chia seeds), and medium-chain triglycerides, particularly C8–C10, while limiting saturated and trans fats.

**Class I; Level B-NR**

**Note:** Evidence suggests that replacing mixed-fat or predominantly saturated-fat sources with polyunsaturated fats may improve the lipid profile, including reductions in LDL cholesterol and total cholesterol. However, direct evidence evaluating the specific combination of monounsaturated fats, omega-3 fatty acids, and medium-chain triglycerides remains limited.

**Question 9: *In adult patients with drug-resistant epilepsy, treated with ketogenic diet therapy, what type of vitamin and mineral supplementation should be administered to prevent nutritional deficiencies and optimize their metabolic status?***

**Recommendation 9**

For adults with drug-resistant epilepsy treated with ketogenic diet therapy, daily supplementation is recommended with a complete multivitamin compound including liposoluble vitamins (A, D, E, K), B-complex vitamins and key minerals such as calcium, magnesium, zinc and selenium, regardless of the type of ketogenic diet therapy used to prevent frequent nutritional deficiencies and maintaining an optimal metabolic status. Commercial ketogenic formulas for ketogenic diet therapy may require less additional supplementation if they are nutritionally complete.

**Class I; Level B-NR**

**Question 10: *In adult patients with drug-resistant epilepsy treated with ketogenic diet therapy, which diagnostic tests should be included in the regular monitoring for early detection of complications (dyslipidemia, hypocalcemia, metabolic acidosis) and the maintenance of optimal ketosis (β-hydroxybutyrate > 2 mmol/L)?***

**Recommendation 10**

In adult patients with drug-resistant epilepsy, treated with ketogenic diet therapy, the recommendation is to adjust the clinical tests monitoring (frequency and type of exam) according to the treatment phase (start, initial maintenance, long-term), the patient’s risk factors and comorbidities. The initial tests include lipid profile, electrolytes, kidney and liver function, vitamin D and β-hydroxybutyrate or ketones in blood and/or in urine. During later stages and on a case-by-case basis, carnitine, uric acid, Vitamin B complex and thyroid evaluation are recommended. A bone density scan is required for patients with an increased risk of osteopenia.

**Class I; Level B-NR**

**Note:** The most frequently reported adverse events associated with ketogenic diet therapy, together with their estimated frequency, suggested monitoring, and management strategies, are summarized in Supplementary Table S2 Ketogenic diet therapy-associated adverse events.

**Review**: Recommendations for follow-up of patients with drug-resistant epilepsy undergoing ketogenic diet therapy and presenting with dyslipidemia Supplementary GPI S1. Good practices recommended to reduce risk (dyslipidemia)

**Question 11: *In patients with drug-resistant epilepsy receiving ketogenic diet therapy, how frequently should laboratory and nutritional assessments be performed to improve seizure control, prevent adverse events, and optimize nutritional status?***

**Recommendation 11**

In adult patients with drug-resistant epilepsy, treated with ketogenic diet therapy, the recommendation is a three-phase clinical, paraclinical and nutritional assessment (lipid profile and kidney function, liver function, electrolytes, vitamin D, carnitine, complete blood count and individualized nutritional assessment):

- Initial Assessment: complete evaluation prior to starting the diet.
- Adaptation (first month): laboratory and nutritional assessments every 1 to 2 weeks to identify early signs and ensure therapeutic ketosis.
- Maintenance: laboratory and nutritional assessments every 3 months for the first year and then every 6 months.

**Class I; Level B-NR**

**Question 12: *In patients with refractory and super-refractory status epilepticus to whom ketogenic diet therapy was prescribed and are going through the acute phase, which diagnostic tests shall be performed to identify or prevent complications?***

**Recommendation 12**

In patients with refractory and super-refractory status epilepticus to whom ketogenic diet therapy was prescribed and who are going through diet initiation, the recommendation is a stepwise approach:

- Before starting complete profile (lipid, kidney, liver, vitamin D*, carnitine*, arterial blood gases, pregnancy test when applicable).
- Induction (days 1–7): monitoring assessments every 8–24 h (glycemia, ketones, electrolytes, gases).
- Stabilization phase: monitoring assessments every 2–3 days.
- Following ICU or recovery: adopt standard ambulatory monitoring.

This approach is intended to align the intensity of follow-up with the patient’s clinical progression.

* The initiation of therapy is not dependent on the report of the result.

**Class I; Level B-NR**

Note: Review PBP 5: Recommendations for adherence with the ketogenic diet therapy in the hospital setting and the ICU and PBP 6: Treatment algorithm with ketogenic diet therapy (KDT) in super-refractory status epilepticus.

## 4. Discussion

***Note***: See Good Practice Item S1. Recommendations for the Follow-Up of Patients with Drug-Resistant Epilepsy Receiving Ketogenic Diet Therapy and Presenting with Dyslipidemia

Available published evidence indicates that ketogenic diet therapy can be implemented safely in adults when delivered within a structured program with appropriate multidisciplinary supervision and monitoring. Rather than being merely a dietary intervention, it constitutes a neurological therapy that requires clearly defined patient-selection criteria and rigorous monitoring, in accordance with international recommendations for the management of adults receiving ketogenic diet therapies [5].

Building on existing international recommendations, this consensus focuses on the practical implementation of ketogenic diet therapy in resource-variable healthcare settings. Whereas current guidance addresses patient selection, treatment initiation, monitoring, supplementation, and adverse-event management, the present document also considers barriers arising from heterogeneous access to trained ketogenic diet teams, laboratory monitoring, commercial ketogenic products, epilepsy surgery, neuromodulation, and standardized pharmacy support [5,9,10,15,20].

Commercial ketogenic formulas may be particularly valuable in hospital and emergency settings because they facilitate the rapid induction of ketosis and precise nutritional control, both of which are important in super-refractory status epilepticus and in patients with poor adherence or limited oral intake [15,16,35]. Similarly, the use of more flexible dietary variants—such as the Modified Atkins Diet, medium-chain triglyceride–based therapies, and the Low Glycemic Index Treatment—recognizes long-term adherence as a key determinant of treatment success and supports a personalized approach rather than a single rigid model [5,10]. This implementation-focused perspective is especially relevant in settings where rapid induction of ketosis, standardized nutrient delivery, and specialized nutritional support may not be consistently available.

The recommendations further emphasize that ketogenic diet therapy may interact with antiseizure medications and other treatments in both hospital and outpatient settings. Systematic medication review should therefore be considered a core component of the treatment protocol and should encompass not only antiseizure medications, but also intravenous solutions, oral liquid formulations, and carbohydrate-containing excipients that may interfere with ketosis or increase the risk of adverse events [15,20].

The consensus also addresses cardiovascular risk as part of the nutritional management of adults receiving ketogenic diet therapy. In patients with dyslipidemia or increased cardiovascular risk, unsaturated fat sources should be prioritized, while saturated and trans fats should be limited. Although available evidence suggests that replacing mixed or predominantly saturated fat sources with polyunsaturated fats may improve selected lipid parameters, direct evidence evaluating the specific combination of monounsaturated fats, omega-3 fatty acids, and medium-chain triglycerides remains limited [19,36].

Beyond the selection of the dietary modality, successful adherence requires a structured protocol that includes patient education, practical tools, intensive support during the first few weeks, and the possibility of transitioning between dietary modalities through progressive adjustments. This approach reinforces the importance of individualized, patient-centered management [5,10,37]. The recommendations also reflect implementation barriers that may be particularly relevant in Colombia, including socioeconomic constraints affecting food selection, variable access to laboratory monitoring and commercial products, and difficulties sustaining long-term adherence.

In addition to seizure reduction, recent studies in adults with drug-resistant epilepsy suggest that ketogenic diet therapies may also be associated with improvements in health-related quality of life [38]. Although patient-reported outcomes were not defined as primary endpoints of this consensus, they are increasingly recognized as relevant to the evaluation of dietary therapies in adult epilepsy and represent an important area for future research [39,40].

The need for specialized multidisciplinary teams suggests that a major implementation challenge lies not only in the availability of evidence but also in the capacity of healthcare services to establish safe and sustainable ketogenic diet therapy programs. Such programs require professional training and appropriate operational adaptations. Accordingly, the proposed clinical and laboratory monitoring supports the early detection, prevention, and management of adverse events and reinforces that treatment safety depends on structured and regular follow-up [5,15].

Several recommendations intentionally align with existing international guidance; their contribution lies in their contextual validation and integration into a comprehensive implementation framework for the Colombian healthcare setting rather than in the novelty of each individual recommendation.

Finally, this consensus supports a shift in the therapeutic positioning of ketogenic diet therapy. It should not be regarded as a last resort, but as an option during intermediate stages of treatment when the goals include seizure reduction, functional improvement, and a reduction in pharmacological burden. Its strategic integration into the management of drug-resistant epilepsy is consistent with current recommendations for the earlier evaluation of non-pharmacological therapies following the failure of two antiseizure medications [3,5,8]. Although this document represents a national consensus, several recommendations may also be applicable to other Latin American healthcare systems with similar structural characteristics and access conditions.

### Limitations

This consensus reflects the experience of Colombian specialists involved in implementing ketogenic diet therapy in adults with drug-resistant epilepsy within healthcare systems characterized by heterogeneous access to advanced epilepsy treatments. The implementation of these recommendations should therefore be adapted to local healthcare resources, infrastructure, and the availability of specialized multidisciplinary teams.

An additional limitation concerns the nature of the available evidence supporting ketogenic diet therapy in adults. The strength of the recommendations was classified using the ACC/AHA framework, which distinguishes the class of recommendation from the level of supporting evidence. Most recommendations were supported by Level B-NR evidence, reflecting the predominance of observational studies and the limited availability of randomized clinical trials involving adults receiving ketogenic diet therapy. No recommendations were classified as expert-opinion-only recommendations (Level C-EO); therefore, the distinction between evidence-based and expert-consensus-informed recommendations is reflected in the grading system applied throughout the manuscript.

## 5. Conclusions

Ketogenic diet therapy is a feasible therapeutic option for adults with drug-resistant epilepsy when implemented through structured protocols and delivered by a trained multidisciplinary team. These recommendations provide practical guidance on patient selection, treatment initiation, monitoring, nutritional supplementation, management of medication–diet interactions, and implementation in both outpatient and acute-care settings, including super-refractory status epilepticus.

By translating international evidence and recommendations into practical guidance adapted to the Colombian healthcare context, this consensus addresses key challenges in the implementation of ketogenic diet therapy in resource-variable settings. Its principal contribution lies in providing a structured operational framework that may help standardize clinical practice, support safer implementation, and reduce the gap between available evidence and routine care.

Although developed in Colombia, several of its principles may be relevant to other Latin American healthcare systems facing similar organizational, socioeconomic, and access-related barriers. Prospective multicenter studies are needed to strengthen the regional evidence on effectiveness, long-term safety, quality of life, treatment adherence, and healthcare resource utilization.

## Data Availability

The original contributions presented in this study are included in the article/Supplementary Materials. Further inquiries can be directed to the corresponding author.

## Supplementary Materials

The following supporting information can be downloaded at: https://www.mdpi.com/article/10.3390/nu18203458/s1, Supplementary Methods S1. Detailed Description of the Modified Nominal Group Technique Consensus Process; Supplementary Table S1. Recommendations by International Organizations on When to Consider Ketogenic Diet Therapy; Supplementary Table S2. Ketogenic Diet Therapy-Associated Adverse Events; Supplementary Table S3. Types of Ketogenic Diet Therapy for Patients with Epilepsy; Good Practice Item S1. Recommendations for the Follow-Up of Patients with Drug-Resistant Epilepsy Receiving Ketogenic Diet Therapy and Presenting with Dyslipidemia; Good Practice Item S2. Absolute and Relative Contraindications to the Initiation of Ketogenic Diet Therapy in Adults with Drug-Resistant Epilepsy; Good Practice Item S3. Recommendations for the Implementation of Ketogenic Diet Therapy in the Outpatient Setting; Good Practice Item S4. Recommendations for the Implementation of Ketogenic Diet Therapy in Hospital and Intensive Care Settings; Good Practice Item S5. Treatment Algorithm for Ketogenic Diet Therapy in Super-Refractory Status Epilepticus; Good Practice Item S6. Paraclinical Tests Required to Identify Contraindications and Optimize the Safety and Efficacy of Ketogenic Diet Therapy in ICU Patients; Supplementary S1. Complete List of References Identified in the Initial Literature Search; Supplementary S2. Complete Search Strategies for Each Clinical Question and Database. References [41,42] are cited in the supplementary materials.

## Editorial Coordination

The editorial coordination of this manuscript was carried out by Integralis HGS, represented by Daniel Rodríguez, MD, and María Stella Salazar, MD.

## Abbreviations

**Abbreviation/Term**	**Definition/Description**
ASDs	Antiseizure Drugs.
β-hydroxybutyrate	Ketone body measures blood as a marker for ketosis (it is not an acronym but is used as a key paraclinical parameter in the recommendations).
CHO	Carbohydrates; present in the management of hypoglycemia: “rapid CHO”.
DBS	Deep Brain Stimulation.
DRE	Drug-resistant epilepsy (refractory or drug-resistant Epilepsy).
EMBASE	Bibliography Database.
GI	Glycemic Index (for instance, carbohydrates with GI < 50).
GLUT1	Glucose Transporter Type 1 Deficiency Syndrome (GLUT1), described in the guidelines as an early onset childhood epilepsy syndrome.
ICU	Intensive Care Unit.
ICT	Information and Communication Technologies.
ILAE	International League Against Epilepsy.
KD	Ketogenic Diet—(described in combinations such as LCT-KD, MCT-KD).
KDT	Ketogenic Diet Therapy
Kidney US	Kidney Ultrasound.
LCT-KD	Long-chain Triglyceride Ketogenic Diet.
LDL	Low Density Lipoproteins (LDL cholesterol).
LGIT	Low Glycemic Index Treatment/Low Glycemic Index Diet—(allows for 40–60 g/day of low glycemic index carbohydrates).
LICE	Italian League Against Epilepsy.
MAD	Modified Atkins Diet.
MCT	Medium-Chain Triglycerides.
MCT-KD	Medium-Chain Triglyceride Ketogenic Diet.
MD	Medical Doctor (as in: “Carlos Andrés Clavijo, MD”).
NAEC	Acronym used as reference for “Guidelines for Specialized Epilepsy Centers” (Neurology, EE. UU., 2024).
ND	Abbreviation for Nutritionist/Dietician.
NG217	NICE code “Epilepsies in children, young people and adults”.
NGT	Nominal Group Technique.
NORSE	New-Onset Refractory Status Epilepticus.
PBP	Acronym used in the document for Good Practices Items (PBP 1, PBP 2, etc.).
PDHD	Pyruvate dehydrogenase deficiency.
PEG	Polyethylene glycol (treatment option for constipation as an adverse event).
PICO	Structured clinical question format (Patient, Intervention, Comparator, Outcome).
PubMed	Bibliography database PubMed.
RNS	Responsive Neurostimulation System.
SRSE	Super-Refractory Status Epilepticus.
VNS	Vagus Nerve Stimulation.
