# Supplementary material for: How to Implement a Ketogenic Diet Therapy in Developing Countries: Colombian Consensus on Ketogenic Diet Therapy Use in the Management of Adults with Drug-Resistant Epilepsy
Source: Nutrients. 2026 Oct 21;18(20):3458. doi: 10.3390/nu18203458 (PMC13708098; doi:10.3390/nu18203458)
Supplement: Supplementary file 1 [file nutrients-18-03458-s001.zip › nutrients-4545883-supplementary.pdf]

nutrients

MDPI

SUPPLEMENTARY MATERIALS FOR THE COLOMBIAN CONSENSUS ON KETOGENIC DIET THERAPY IN ADULTS WITH DRUG-RESISTANT EPILEPSY

- **Supplementary Methods S1**. Detailed Description of the Modified Nominal Group Technique Consensus Process
- **Supplementary Table S1.** Recommendations by International Organizations on When to Consider Ketogenic Diet Therapy
- **Supplementary Table S2**. Ketogenic Diet Therapy-Associated Adverse Events
- **Supplementary Table S3.** Types of Ketogenic Diet Therapy for Patients with Epilepsy
- **Good Practice Item S1.** Recommendations for the Follow-Up of Patients with Drug-Resistant Epilepsy Receiving Ketogenic Diet Therapy and Presenting with Dyslipidemia
- **Good Practice Item S2.** Absolute and Relative Contraindications to the Initiation of Ketogenic Diet Therapy in Adults with Drug-Resistant Epilepsy
- **Good Practice Item S3**. Recommendations for the Implementation of Ketogenic Diet Therapy in the Outpatient Setting
- **Good Practice Item S4**. Recommendations for the Implementation of Ketogenic Diet Therapy in Hospital and Intensive Care Settings
- **Good Practice Item S5**. Treatment Algorithm for Ketogenic Diet Therapy in Super-Refractory Status Epilepticus
- **Good Practice Item S6**. Paraclinical Tests Required to Identify Contraindications and Optimize the Safety and Efficacy of Ketogenic Diet Therapy in ICU Patients
- **Supplementary S1**. Complete List of References Identified in the Initial Literature Search
- **Supplementary S2.** Complete Search Strategies for Each Clinical Question and Database

Supplementary Methods S1: Detailed Description of the Modified Nominal Group Technique Consensus Process

S1.1. Expert Panel

Fourteen Colombian experts participated in the project: 11 neurologist-epileptologists and three nutritionists. Participants were selected based on their expertise, academic contributions, research experience, and clinical experience in the management of patients with drug-resistant epilepsy using ketogenic diet therapy.

S1.2. Consensus Methodology

En 2.1. Expert Panel and Consensus Design.

The project comprised nine phases conducted using a modified Nominal Group Technique supported by information and communication technology tools.

The Nominal Group Technique comprises four core components:

1. Independent generation of ideas or responses.
2. Review of peer contributions, with an opportunity to revise the initial responses.
3. Clarification of concepts, arguments, or positions.
4. Structured voting.

The modified format enabled remote and asynchronous participation. Individual response generation and peer review were conducted through a web-based platform, whereas clarification and voting were performed synchronously through videoconferencing and online voting tools.

This modified approach preserved the essential characteristics of the Nominal Group Technique, including independent response generation and structured voting, while allowing anonymity during the initial phases and facilitating the participation of a larger expert group. Unlike the Delphi method, consensus was reached directly by the participating experts through iterative voting rounds governed by predefined agreement thresholds.

Initial response generation, peer review, and asynchronous voting were conducted through a purpose-built web-based platform developed specifically for this consensus process, whereas

clarification and synchronous voting were performed through videoconferencing and online voting tools.

S1.3. Phase 1: Standardization of the Clinical Questions

The clinical questions addressed by the consensus group were standardized using the PICO format.

S1.4. Phase 2: Literature Search and Study Selection

This phase included the development of the search strategies and the selection of relevant articles. Articles published from 2015 onward were considered because most of the evidence specifically addressing ketogenic diet therapy in adults is recent, whereas earlier evidence had been evaluated in previous systematic reviews, including Cochrane reviews. During manuscript revision, selected references were updated through June 2026 to incorporate more recent evidence; the original database searches were not rerun.

Search Strategy

Based on the clinical questions formulated by the Ketogenic Diet Therapy Consensus Group, search strategies were developed according to the PICO structure. Searches were conducted for all clinical questions in PubMed and EMBASE. For each question, the searches were conducted by two independent reviewers, and the resulting strategies were consolidated for each database. The eligibility of each identified record was assessed manually.

search strategies for each clinical question and database are provided in Supplementary Annex [X]. The clinical questions were structured according to the PICO format. Search strategies were developed for each question, and literature searches were conducted in PubMed and EMBASE. The complete list of references identified in the initial literature searches, organized by clinical question, is provided in Supplementary S1. Given its length, the complete list is presented as supplementary material rather than reproduced in the main manuscript.

Study Selection

https://doi.org/10.3390/nu18203458

Articles published from 2015 onward were considered because most of the relevant evidence in adult populations is recent and earlier evidence had been evaluated in previous systematic reviews, including Cochrane reviews.

Eligible study designs included:

- systematic reviews;
- randomized clinical trials;
- non-randomized trials;
- cohort studies;
- case-control studies; and
- case series.

Disagreements between reviewers were resolved by consensus. When necessary, a third reviewer participated in the decision-making process. This reviewer was a psychologist with a master's degree in Clinical Epidemiology and extensive experience in evidence appraisal and scientific research.

The methodological quality and risk of bias of the included evidence were assessed according to study design:

- clinical practice guidelines were evaluated using the AGREE II instrument;
- systematic reviews were assessed using ROBIS;
- randomized controlled trials were assessed using the Cochrane Risk of Bias 2 tool;
- non-randomized studies of exposures were assessed using ROBINS-E; and
- cohort and case-control studies were evaluated using the Newcastle–Ottawa Scale.

S1.5. Phase 3: Definition of the Maximum Number of Options

For each clinical question, experts were required to select only one option as the proposed recommendation.

S1.6. Phase 4: Generation of Individual Responses

https://doi.org/10.3390/nu18203458

Each expert received the clinical questions, instructions for submitting the proposed responses through the web-based application, and the full text of the articles selected during Phase 2.

Experts were also instructed to submit any additional reference that they considered relevant and that had not been included in the application.

S1.7. Phase 5: Evidence Assessment and Grading

The consensus used the classification framework of the American College of Cardiology Foundation/American Heart Association Task Force on Practice Guidelines.

This phase included assessment of the methodological quality of the articles comprising the body of evidence for each clinical question. The framework was selected because it allows the class of recommendation and the level of supporting evidence to be assessed separately and is applicable to clinical consensus processes based on heterogeneous bodies of evidence.

S1.8. Phase 6: Individual Review of Responses

Using the web-based application, each participant reviewed the individual responses submitted by the other experts for each clinical question.

After reviewing the responses of the other panel members, participants could amend their own previously submitted answers.

S1.9. Phase 7: Analysis and Synthesis of Responses

The final responses submitted by the experts were reviewed and synthesized using affinity matrices.

Responses that did not comply with the required structure of a recommendation were excluded. Responses with similar content, wording, and organization were consolidated into individual recommendation options.

During the synthesis phase, the Integralis methodological team reviewed the final responses. Responses were excluded when they did not directly address the corresponding clinical question, were not formulated as a recommendation statement, or were too incomplete or ambiguous to be

https://doi.org/10.3390/nu18203458

converted into a recommendation without altering their intended meaning. Responses with similar clinical content were consolidated using affinity matrices.

The resulting options were subsequently submitted to the experts for voting during Phase 8.

S1.10. Phase 8: asynchronous voting.

During Phase 8, the experts completed an asynchronous voting process through the web-based platform.

S1.11. Phase 9: synchronous clarification and final voting.

During Phase 9, the panel participated in two synchronous videoconference meetings. The recommendation options were discussed, questions regarding their wording were addressed, and voting was conducted on the recommendation statements, their class of recommendation, and their level of evidence.

S1.11. Summary Flow Chart for modified NGT method used [1–3].

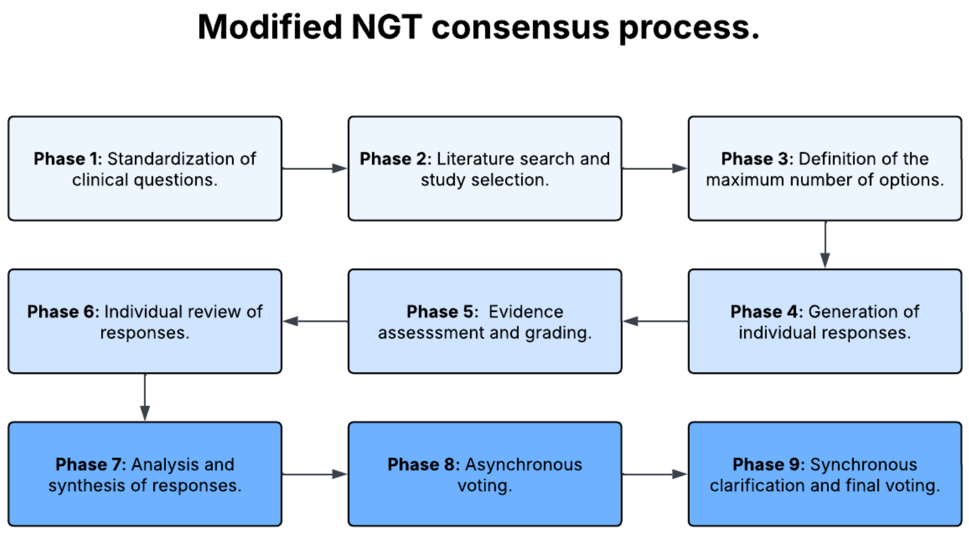

Supplementary Table S1: Recommendations by International Organizations on Time to Consider Ketogenic diet therapy

Guideline / Organization	Scope	Position of Ketogenic diet therapy (KDT)	Relation to Surgery / Neuromodulation	Key Implementation Considerations
National Institute for Health and Care Excellence NICE NG217 (UK, 2022; update 2025) [4]	Comprehensive guideline for diagnosis and treatment of epilepsy in children, young people, and adults	KDT recommended as a non-pharmacological option in drug-resistant epilepsy when other treatments are ineffective or not appropriate; particularly supported in selected epilepsy syndromes with childhood onset	Presented alongside epilepsy surgery and VNS as tertiary-care options for DRE management	Should be initiated and supervised in tertiary specialist centers
National Association of Epilepsy Centers NAEC / *Neurology* (USA, 2024) [5]	Standards for organization and service portfolio of specialized epilepsy centers	Recognizes KDT as an effective treatment option in adults, with better tolerability reported for MAD and LGIT compared with classical KDT	Included within comprehensive services expected in specialized epilepsy centers together with surgery and neuromodulation programs	Requires structured protocols and participation of trained dietitians and epileptologists
International League Against Epilepsy ILAE Surgical Commission (2022) [6]	Recommendations for management of drug-resistant epilepsy and referral for surgical evaluation	KDT considered part of the non-pharmacological therapeutic armamentarium within tertiary-level management	Strong recommendation for early referral for surgical evaluation after confirmation of DRE; KDT positioned as complementary option within advanced care pathways	Reinforces early tertiary referral and multidisciplinary evaluation
Italian League Against Epilepsy LICE (2023) [7]	Practical recommendations for implementation of ketogenic therapies	Provides structured protocols for classical KDT, MCT, MAD, and LGIT in DRE patients, including adults	Positions KDT as a structured therapeutic option integrated within multidisciplinary epilepsy care	Emphasizes patient education, metabolic monitoring, and individualized dietary selection

International Adult KDT Consensus (2021) [8]	Consensus focused specifically on ketogenic diet therapy in adults with epilepsy	Defines candidate selection criteria, monitoring strategies, adverse-event management, supplementation, and adherence considerations	Positions KDT within therapeutic algorithm after failure of antiseizure medications and alongside other non-pharmacological options	Provides practical guidance for hospital vs ambulatory initiation and long-term follow-up

Supplementary Table S2: Ketogenic diet therapy-associated adverse events [9,10]

**System**	**Complication**	**Estimated Frequency**	**Mechanism / risk**	**Monitoring**	**Suggested management**
Gastrointestinal	Constipation	9–40%	Fiber reduction	Bowel movement frequency	Fiber, PEG
Gastrointestinal	Nausea/vomiting	Frequent at the start	High lipid burden	Clinical	Divide meals
Gastrointestinal	Abdominal pain/reflux	Occasional	Gastric irritation	Clinical	Adjust the formula
Metabolic	Metabolic acidosis	20–30% (SRSE)	Increased ketonic bodies	Bicarbonate, pH	Oral bicarbonate oral/IV
Metabolic	Hypoglycemia	13–27%	Gluconeogenesis suppression	Glycemia	Rapid CHO, adjust fats
Metabolic	Hyperlipidemias	Up to 40%	Elevated saturated fat	Lipid profile	Reduce animal fat
Metabolic	Weight loss	7–25%	Calorie deficit	Weight	Adjust kcal

https://doi.org/10.3390/nu18203458

Renal / Urinary	Kidney stones	<10%	Hypercalciuria, Ketosis	Kidney US	Citrate, hydration

Source: *Prepared by the author, Carlos Andrés Clavijo, MD.*

Classification criteria: Early: Usually presents over the first 2–4 weeks; Late: Frequently after the first month or associated with an accumulation of deficits or metabolic adaptation.

Estimated frequencies are derived from pooled data reported across multiple clinical studies and reviews. The cited references support the type, mechanisms, monitoring, and management of adverse events associated with ketogenic diet therapy.

https://doi.org/10.3390/nu18203458

Supplementary Table S3 - Types of ketogenic diet therapy for patients with epilepsy [11,12]

Several ketogenic dietary therapy variants are available for adults with drug-resistant epilepsy, differing in ketogenic intensity, flexibility, tolerability, and implementation complexity

Type of therapy	Brief description	Use in epilepsy	Indications for adults
Classic ketogenic diet (4:1 or 3:1)	High in fat, very low in carbohydrates. Fixed fat: protein + carbohydrates ratio. Requires strict supervision.	Highly effective in refractory epilepsy. Is the historical therapeutic standard.	Requires initial hospitalization in most cases. Useful in refractory status epilepticus (RSE) and frequent seizures. Lower compliance in adults.
Modified Atkins Diet (MAD)	Most flexible variant. Limits carbohydrates to 10–20 g/day without strict count of fats or proteins.	Comparable efficacy to the classic in adults; improved compliance.	Ideal in the ambulatory setting. High applicability in active adults or in adults with comorbidities.
Low Glycemic Index Treatment (LGIT)	Allows 40–60 g/day of low GI carbohydrates (<50). Produces milder ketosis and improved glycemic stability.	Moderate efficacy: useful when other diets are not well tolerated. More heterogeneous evidence in adults.	Elderly people, patients with metabolic risk or requiring improved daily tolerance.
MCT diet (Medium Chain Triglycerides)	In adults, the safest/better tolerated MCT ratio is ~20–30 % of energy, and when used as the main lipid source (higher percentages) there is an increased risk of gastrointestinal events: diarrhea, bloating, nausea at high doses (>30% TCV)	Limited evidence in adults. Similar global efficacy to the classic, but with more gastrointestinal events.	Cases with malabsorption, intolerance to very restrictive diets, or need for increased flexibility.

Source: *Prepared by the author, Nataly Rincónn Franco ND* [8,16–18]

The classic ketogenic diet, the modified Atkins, the low glycemic index, and the medium chain triglycerides diet, have all proven to be effective in drug-resistant epilepsy. Systematic reviews and the recent guidelines fail to contribute to high quality evidence

https://doi.org/10.3390/nu18203458

showing consistent differences in efficacy among them; therefore, the selection is usually based mainly on tolerability, comorbidities profile and logistics feasibility.

https://doi.org/10.3390/nu18203458

GOOD PRACTICE ITEMS (GPI)

Good Practice Item S1. Recommendations for the Follow-Up of Patients with Drug-Resistant Epilepsy Receiving Ketogenic Diet Therapy and Presenting with Dyslipidemia [3,22]

- Assess the causes of dyslipidemia
- Assess the lipid profile levels – should not double the maximum allowable limit
- Select the profile of fats administered: prevalence of sources such as mono and polyunsaturated, restriction of saturated fats and exclusion of trans fats.
- Ensure adequate supply of fiber requirements since this helps to lower LDL cholesterol.

Hyperlipidemia is a frequent condition in people managed with ketogenic diet therapy and mostly develops when the classic ketogenic diet is used, with a high percentage of saturated fats. One of the strategies recommended to lower the risk is to prefer the Modified Atkins diet, including a balanced profile of unsaturated fats. The use of medium chain triglycerides has also shown to reduce the risk.

https://doi.org/10.3390/nu18203458

Good Practice Item S2. Absolute and Relative Contraindications to the Initiation of Ketogenic Diet Therapy in Adults with Drug-Resistant Epilepsy [3, 4, 23]

TYPE OF CONTRAINDICATION	CLINICAL CONDITION	COMMENTARY / RISK MECHANISM	IS IT POSSIBLE TO USE IT WITH CAUTION?
**Absolute**	Fatty acid metabolism deficit (ex. carnitine, CPT I/II, acyl-CoA dehydrogenases). Concomitant use with propofol [24]	Inability to develop ketone bodies; risk of hypoglycemia, acidosis and organ damage.	No
**Absolute**	Pyruvate carboxylase deficiency	Severe gluconeogenesis disfunction; risk of severe hypoglycemia.	No
**Absolute**	Acute intermittent porphyria	Ketosis / fasting may trigger a porphyria attack.	No
**Absolute**	Acute active pancreatitis	A high lipid intake may further deteriorate pancreatic inflammation.	No
**Absolute**	Advanced liver disease	The liver is essential for the production of ketone bodies.	No
**Relative**	Chronic, moderate or severe kidney failure (GF < 30 ml/min)	Risk of acidosis, electrolyte imbalance, lipid load intolerance.	Yes, with nephrology follow-up
**Relative**	Type 1 Diabetes Mellitus	Risk of diabetic ketoacidosis in the absence of rigorous follow-up.	Yes, with intensive endocrine follow-up
**Relative**	Heart failure (NYHA III–IV)	Risk of metabolic intolerance in advanced disease.	Yes, with cardiology assessment

**Relative**	Severe chronic lung disease	Potential theoretical risk of acidosis / metabolic imbalances.	Yes, with respiratory monitoring and support
**Relative**	Pregnancy and lactation	Limited human data; maternal-fetal nutritional risk.	Yes, milder modality and under extreme surveillance
**Relative**	Active cancer treatment	Risk dependent on the type of tumor; some emerging evidence.	Yes, case by case
**Relative**	Geriatric frailty, polypharmacy, cognitive decline	Higher risk of dehydration, compliance errors, side effects.	Yes, with intensive support
**Relative**	Preoperatory / impending surgery	Surgical stress demands glucose; risk of acidosis.	Yes, consider temporary discontinuation
**Relative**	Active eating disorders	Risk of exacerbating restrictive or compulsive patterns.	Yes, only with mental health support

Source: Prepared by the author, Nataly Rincón Franco ND.

Notes:

- This table has been updated based on the original article by Kossoff et al., including evidence recently published between 2022 and 2024. Some conditions previously considered as absolute contraindications have been classified as relative, allowing for management under individualized protocols and with multidisciplinary monitoring.
- This table focuses on the implementation of ketogenic diet therapy in adult population with epilepsy. Hence, childhood onset epileptic syndromes with little prevalence in adults are not included (such as Angelman,

https://doi.org/10.3390/nu18203458

Dravet, Doose, among other syndromes). If necessary, these syndromes are available for consultation in specialized pediatric guidelines.

https://doi.org/10.3390/nu18203458

Good Practice Item S3. Recommendations for the Implementation of Ketogenic Diet Therapy in the Outpatient Setting [13,14]

	**JUSTIFICATION**	**RECOMMENDATION**
**Pre-Assessment**	• Before starting the ambulatory ketogenic diet therapy:<br>• Specialist evaluation to request and review the baseline laboratory tests.<br>• Initial nutritional assessment to identify any risk factors that may compromise compliance or the health status of the patient in the long term.	• Request and review baseline laboratory tests.<br>• Complete nutritional assessment (dietary and nutritional history, habits, anthropometrics, etc.)
**Selection and initiation of diet**	The type of diet selection should be individualized. However, evidence shows that the Modified Atkins diet should be prioritized.	• Start with the Modified Atkins Diet type:<br>- Fats ≈ 65% of the total caloric value<br>- Carbohydrates ≈ 10% of the total caloric value<br>- Proteins 0.8 – 1.2 g/Kg/day<br>• Start gradually and with no previous fasting.<br>• Intensive patient and family education about the different fat sources, constipation management and the importance of hydration.
**Prevention of complications**	For improved compliance, the most frequent complications should be prevented. To this end, micronutrient supplementation is important (commercial formulas include micronutrients, but it is necessary to check if the dose is sufficient) and ensuring adequate hydration.	• Consider the following supplements (individualize according to the case)<br>- Complete multivitamin supplement (with no added carbohydrates)<br>- Calcium + vitamin D3.<br>- Magnesium or potassium citrate according to symptoms or risk.

https://doi.org/10.3390/nu18203458

		- Carnitine if deficient, or in the presence of fatigue.<br>• Hydration recommendation: (≥2 L/day).
**Monitoring and follow-up**	Follow-up visits with the treating specialist and with the nutritionist are essential to achieve ketogenic treatment compliance.	• Clinical monitoring: at one month, at 3 months, at 6 months and then every 6–12 months if the patient is compliant.<br>• Laboratory monitoring test at 3 and 6 months.<br>• Ketones monitoring with a goal of β-hydroxybutyrate of 1–3 mmol/L.
**Evaluation and progressio n**	The ketogenic diet is dynamic and should be adjusted according to the patient's condition.	• Assess response at 3 months (no not discontinue earlier except for severe intolerance).<br>• Maintain if there is a reduction in seizures of ≥50% or cognitive/behavioral improvement.<br>• If there is no response, consider progression to classic ketogenic diet (2:1–3:1) ensuring protein intake or adding MCT.

Source: *Prepared by the author, Lina Marcela Ramírez Hurtado ND.*

- *The Modified Atkins Diet, or a ketogenic ratio of 1:1, 2:1, 2.5:1 meets the protein requirements of the adult population. Higher ketogenic ratios should be considered only for a limited time and with adequate monitoring to prevent nutrient (protein) deficit.*
- *The caloric intake must meet the nutritional needs of each patient.*
- *Ensure compliance with the requirements of vitamins, minerals, fiber and fluids to prevent undesirable side events.*

Good Practice Item S4. Recommendations for the Implementation of Ketogenic Diet Therapy in Hospital and Intensive Care Settings[26]

I. Selection of candidate patients for ketogenic diet therapy

- Continuous video EEG monitoring.
- Use inclusion and exclusion criteria.
- Request and check the necessary laboratory tests to start the ketogenic diet therapy.
- Tame anthropometric measurements: weight, size.

Identify any absolute or relative contraindications and to make the necessary changes for optimal performance of the therapy.

- The recommendation is to start with ketogenic ratios of 4:1 or 3:1
- Caloric intake: 25-30 cal/kg, hypercaloric diets may affect ketosis.
- Use of ketogenic formulas with adequate fat, mineral and fiber profiles.
- Restriction of dextrose solutions and carbohydrate-containing medications.
- Avoid the use of propofol.
- Ensure adequate hydration.
- Constant monitoring of BHB (beta-hydroxybutyrate) levels and glycemia.
- Consider using carnitine 10-50 mg/kg/day in patients receiving valproate or with elevated serum triglycerides.

II. Starting a ketogenic diet therapy

- Minimize carbohydrates in medications, parenteral solutions and IV fluids with the support of pharmacy.
- Discontinue propofol infusion 24 before initiating the ketogenic diet therapy to avoid propofol infusion syndrome, which is probably lethal, characterized by metabolic acidosis.
- Establish a safe feeding route to start enteral nutritional support with ketogenic diet therapy.

https://doi.org/10.3390/nu18203458

- Consult with a nutritionist to design the nutritional plan according to the nutritional status of the patient.
  - Defining the type of ketogenic diet: in patients with SRSE the recommendation is to start with a Classic Ketogenic Diet in ratios of 4:1 or 3:1. In patients at high nutritional risk, the recommendation is to start with a 3:1 ratio (Calculation shown in Annex 1).
  - Establish the nutritional requirements:
    - Calorie requirement: the recommendation is to start with an intake of 25-30 Kcal/kg/day. Hypercaloric diets may impact ketosis.
    - Protein requirement: the recommendation for the critical patient is to establish a target intake of 1.2 to 2.0 g/kg/day. However, at the start of the therapy with the Classic Ketogenic Diet in a 4:1 or 3:1 ratio, a maximum of 0.5 – 0.8 g of protein per kg of weight will be achieved; this is the necessary intake to favor ketosis.
    - Fat requirement: this is the most important component of the ketogenic diet, with over 80% of the calories obtained from lipids in the mentioned ketogenic ratios. The recommendation is to strive for an adequate intake of monounsaturated and polyunsaturated fatty acids, as well as an MCT which does not exceed 30% of the total caloric intake.
    - Carbohydrate requirement: the recommendation is an intake of 10 ato20 g/day, representing approximately 3 % of the total caloric intake.
    - Micronutrients recommendation: adequate micronutrients should be provided to meet the recommended daily intake according to the age and gender of the patient. Consider the use of carnitine 10-50 mg/kg/day in patients receiving valproate o with elevated serum triglycerides.
    - Water requirement: adequate hydration must be ensured, recommending an intake of 25–30 mL/kg/day of total water (including IV fluids, nutrition and medications).
  - Select the commercial formula to initiate ketogenic diet therapy:
    - If the patient is undergoing enteral nutrition support with catheter, the recommendation is to replace the enteral formula with a ketogenic formula with a 4:1 ratio, adequate fat profile, vitamins, mineral and

fiber; starting with 50% of the estimated calorie requirements during the first 24 hours and then progressing according to tolerance towards the target requirements.

- If the patient is fasting and there is no risk of refeeding syndrome, the recommendation is to administer enteral nutritional support with ketogenic formula in a 4:1 ratio, via a feeding tube, with an adequate fat, vitamins, minerals and fiber profile. Start with 33% - 50% of the estimated calorie requirements during the first 48 hours and then escalate according to tolerance to reach the target requirements.

Note: In case the initiation of ketogenic diet therapy is established with a 3:1 ratio, the recommendation is to select a ketogenic formula with a 4:1 ratio and make calculations for additional protein intake that should be provided via a protein module to reach such ratio. (Example in Annex 1).

Ketogenic diet therapy maintenance

- Monitor capillary glucose every 4 hours and administer a dextrose IV bolus (12.5 g) every 20 minutes if <50 mg/dL.
- Check urinary ketones and serum β-hydroxybutyrate every 12 hours (urine acetoacetate ≥40 mg/dl and/or serum β-hydroxybutyrate ≥2 mmol/l); after 48 hours, monitor the metabolic status (if needed, supplement bicarbonate to maintain a serum bicarbonate level >18 mEq/L).
- Continue adjusting the sedative medication to suppress seizures for 72 hours (with no additional changes in antiepileptic drugs, unless the medical team considers it necessary).
- Gradually reduce sedatives after 72 hours:
    - If this approach fails, reinitiate the anesthetic agent and try again in one week's time.
    - If after two weeks the ketogenic diet therapy fails to succeed, the recommendation is to discontinue the treatment.
    - If the therapy is successful after reducing the sedatives, the recommendation is to continue with the ketogenic formula 4:1 and gradually withdraw the initial ketogenic ratio to adequately meet the patient's protein requirements.

https://doi.org/10.3390/nu18203458

Ketogenic diet therapy transition

- After resolution of the SRSE and upon identifying a clinical improvement of the patient, the recommendation is to transition to the oral route.
- If the patient is being fed by mouth, the recommendation is to initiate the modified Atkins diet (MAD) with 20 g net of carbohydrates per day, high fat content (60% of the total caloric value) and adequate protein intake to reach a urine ketone target of ≥40 mg/dL.
  - In addition to MAD, other ketogenic diets have been described as beneficial for controlling seizures, including the ketogenic diet with MCT, the Low Glycemic Index Diet or the Modified Ketogenic Diet. The trained nutritionist shall select the best option for the patient, in accordance with the nutritional assessment and an individualized approach.
- Record urine ketones twice per week, weekly body weight, and daily frequency of seizures.
- Establish ambulatory follow-up in one month and request laboratory follow-up tests.
- **Note:** Recommendations are based on published evidence and expert consensus adapted to institutional ICU protocols.

https://doi.org/10.3390/nu18203458

Good Practice Item S5. Treatment Algorithm for Ketogenic Diet Therapy in Super-Refractory Status Epilepticus [11, 26, 27]

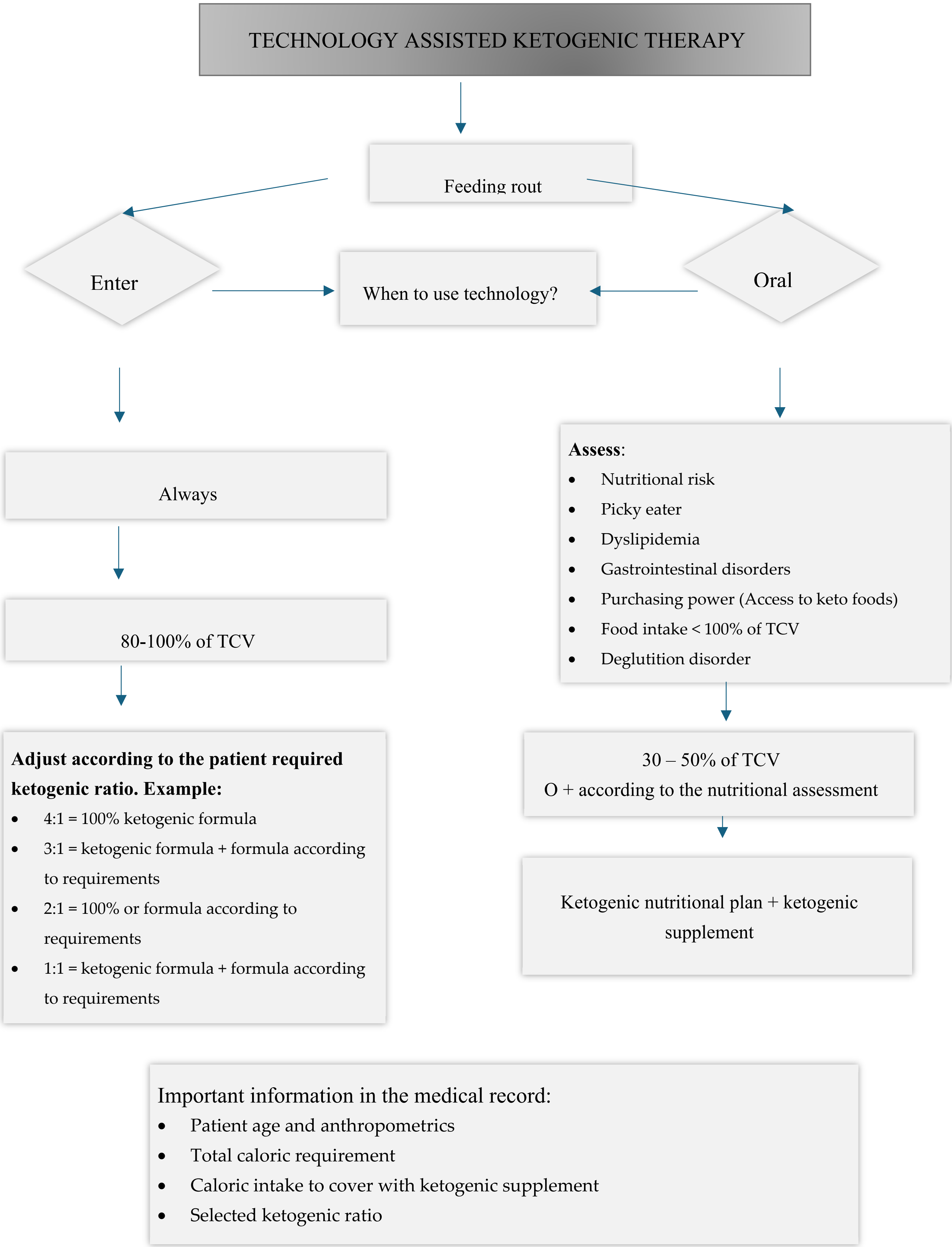

https://doi.org/10.3390/nu18203458

Source: *Prepared by the authors, Walter González MD, and Angélica Lizcano MD.*

https://doi.org/10.3390/nu18203458

Good Practice Item S6. Paraclinical tests required to identify any contraindications and optimize safety and efficacy of treatment with ketogenic diet therapy of an ICU patient

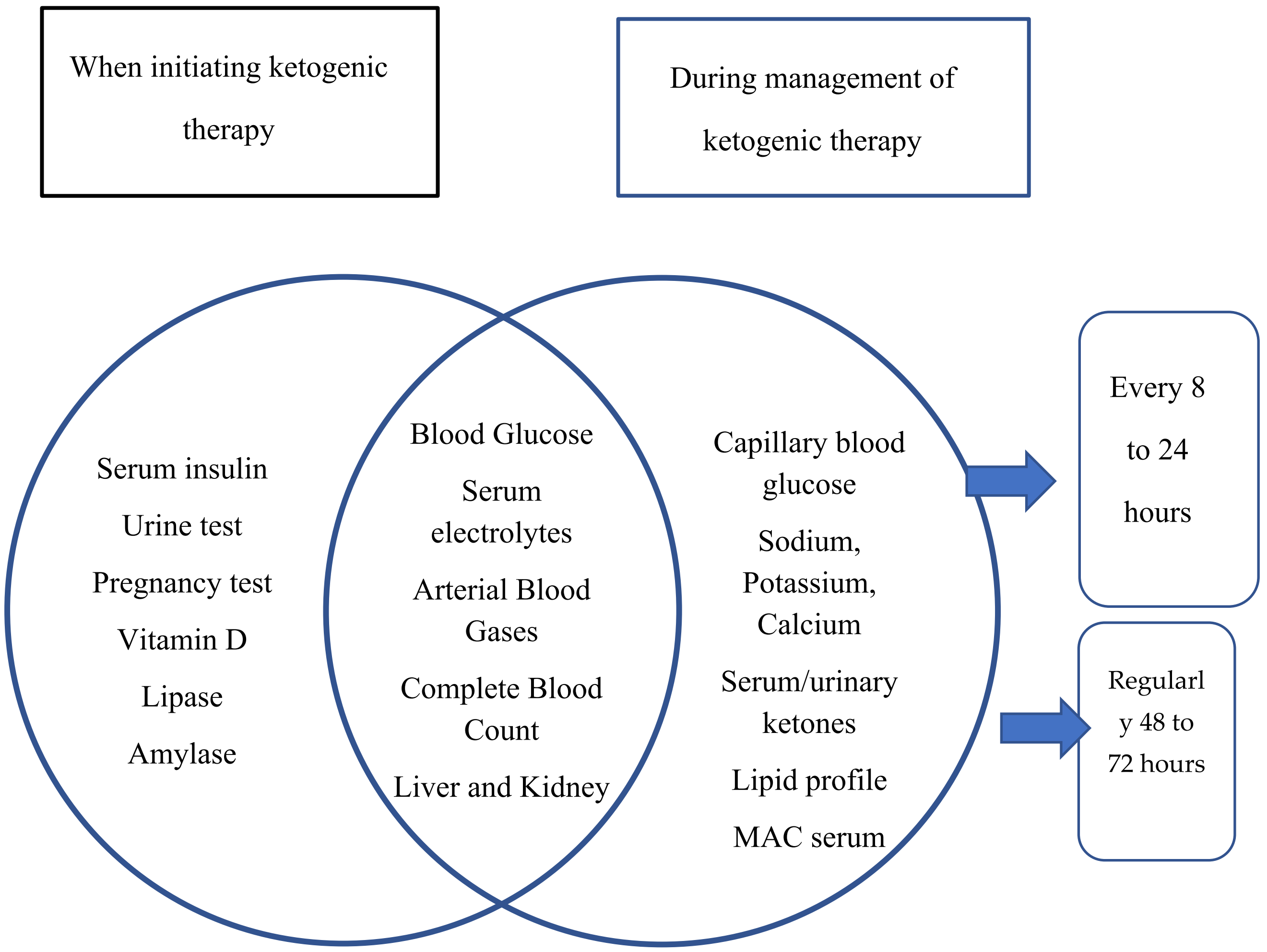

Note: Additional laboratory tests may be considered based on clinical context and institutional protocol.
Source: *Prepared by the author, Lyda Villamil-Osorio MD.*

https://doi.org/10.3390/nu18203458

Supplementary S1: Complete List of References

Question	Reference	URL
1	H. Kossoff, 2011	https://firebasestorage.googleapis.com/v0/b/xpert-opinion-3487b.appspot.com/o/TCA%2FParticipants%2F67f45fe83e26a1ee2ad84355%2F%20H.%20Kossoff%2C%202011?alt=media&token=43052444-c8ad-4ef0-adbf-e8414f9ec8be
1	Alanis, 2024	https://firebasestorage.googleapis.com/v0/b/xpert-opinion-3487b.appspot.com/o/TCA%2FBase%2FAlanis_2024.pdf?alt=media&token=f0cda3d6-dd1f-482e-b9ec-1292b244aa63
1	Ballesteros, 2024	https://firebasestorage.googleapis.com/v0/b/xpert-opinion-3487b.appspot.com/o/TCA%2FBase%2FBallesteros_2024.pdf?alt=media&token=7b2c4403-da93-4ccc-9e53-b8d901fd9a57
1	Budiputra, 2023	https://firebasestorage.googleapis.com/v0/b/xpert-opinion-3487b.appspot.com/o/TCA%2FBase%2FBudiputra_2023.pdf?alt=media&token=48f23c04-54b5-4b2e-835d-b24ad081f9c4
1	Cervenka, 2021	https://firebasestorage.googleapis.com/v0/b/xpert-opinion-3487b.appspot.com/o/TCA%2FParticipants%2F67f45f9a3e26a1ee2ad672bc%2FCervenka%2C%202021?alt=media&token=017116d1-55a9-4968-ae92-f42a85b67a5c
1	Erkent, 2023	https://firebasestorage.googleapis.com/v0/b/xpert-opinion-3487b.appspot.com/o/TCA%2FBase%2FErkent_2023.pdf?alt=media&token=2d2ed0e8-cc0c-4930-a401-a30e86b7df23
1	Falco, 2019	https://firebasestorage.googleapis.com/v0/b/xpert-opinion-3487b.appspot.com/o/TCA%2FBase%2FFalco_2019.pdf?alt=media&token=31f131a7-ff73-42d6-90f8-966b04ebe97d
1	Griffen, 2023	https://firebasestorage.googleapis.com/v0/b/xpert-opinion-3487b.appspot.com/o/TCA%2FBase%2FGriffen_2023.pdf?alt=media&token=6e147722-7199-40d1-a319-0396e9242e41
1	Koh, 2022	https://firebasestorage.googleapis.com/v0/b/xpert-opinion-3487b.appspot.com/o/TCA%2FBase%2FKoh_2022.pdf?alt=media&token=45adcc46-6f7b-436a-a4c2-ba7e3e53725f
1	Li, 2024	https://firebasestorage.googleapis.com/v0/b/xpert-opinion-3487b.appspot.com/o/TCA%2FBase%2FLi_2024.pdf?alt=media&token=585e0d1d-f0fb-4107-bc94-ac6771337578
1	Martin, 2017	https://firebasestorage.googleapis.com/v0/b/xpert-opinion-3487b.appspot.com/o/TCA%2FBase%2FMartin_2017.pdf?alt=media&token=1780b55d-0b82-48ac-8953-29e0f889d0cf
1	Martin, 2020	https://firebasestorage.googleapis.com/v0/b/xpert-opinion-3487b.appspot.com/o/TCA%2FBase%2FMartin_2020.pdf?alt=media&token=7dca3dd2-72d9-40db-945b-838e61e0be84
1	McDonald, 2018a	https://firebasestorage.googleapis.com/v0/b/xpert-opinion-3487b.appspot.com/o/TCA%2FParticipants%2F67f45fe83e26a1ee2ad84355%2FMcDonald%2C%202018a?alt=media&token=5851f761-1e7b-44fa-906d-1581d52f2257
1	Mendonca, 2024	https://firebasestorage.googleapis.com/v0/b/xpert-opinion-3487b.appspot.com/o/TCA%2FBase%2FMendonca_2024.pdf?alt=media&token=7f51734d-3497-440b-bc89-6ebe4774658d
1	Quiroga, 2022	https://firebasestorage.googleapis.com/v0/b/xpert-opinion-3487b.appspot.com/o/TCA%2FBase%2FQuiroga_2022.pdf?alt=media&token=608ec8e8-217c-45ed-9218-96b373aebb4d

1	Schoeler, s.f	https://firebasestorage.googleapis.com/v0/b/xpert-opinion-3487b.appspot.com/o/TCA%2FBase%2FSchoeler_s.f.pdf?alt=media&token=edf3ff3d-dc1d-453d-99d3-1b74c6e76e1f
1	Zhang, 2024	https://firebasestorage.googleapis.com/v0/b/xpert-opinion-3487b.appspot.com/o/TCA%2FBase%2FZhang_2024.pdf?alt=media&token=62bababc-eedb-4ba9-82b9-37fc09dfe556
2	Camoes, 2021	https://firebasestorage.googleapis.com/v0/b/xpert-opinion-3487b.appspot.com/o/TCA%2FBase%2FCamoes_2021.pdf?alt=media&token=63673763-1928-4e6b-bcfb-9c51f4c9640f
2	Cervenka, 2017	https://firebasestorage.googleapis.com/v0/b/xpert-opinion-3487b.appspot.com/o/TCA%2FBase%2FCervenka_2017.pdf?alt=media&token=b135e7aa-c6cd-48a4-b34d-3af7b2ea31a9
2	Hanin, 2024	https://firebasestorage.googleapis.com/v0/b/xpert-opinion-3487b.appspot.com/o/TCA%2FBase%2FHanin_2024.pdf?alt=media&token=2ee9cba0-463c-4769-b26d-dffa75132fa0
2	Kaul, 2020	https://firebasestorage.googleapis.com/v0/b/xpert-opinion-3487b.appspot.com/o/TCA%2FBase%2FKaul_2020.pdf?alt=media&token=89540e82-35af-47a8-a79f-e8231a06979d
2	Kaul, 2022	https://firebasestorage.googleapis.com/v0/b/xpert-opinion-3487b.appspot.com/o/TCA%2FBase%2FKaul_2022.pdf?alt=media&token=3cf2d0bb-808e-497a-9adb-377126edc0c3
2	Koh, 2022	https://firebasestorage.googleapis.com/v0/b/xpert-opinion-3487b.appspot.com/o/TCA%2FBase%2FKoh_2022.pdf?alt=media&token=45adcc46-6f7b-436a-a4c2-ba7e3e53725f
2	Park, 2019	https://firebasestorage.googleapis.com/v0/b/xpert-opinion-3487b.appspot.com/o/TCA%2FBase%2FPark_2019.pdf?alt=media&token=733a72de-f443-41e5-ba2b-fc8e00613e7f
2	Prasoppokakorn, 2019	https://firebasestorage.googleapis.com/v0/b/xpert-opinion-3487b.appspot.com/o/TCA%2FBase%2FPrasoppokakorn_2019.pdf?alt=media&token=1cb9a91d-4736-469c-a2b6-f982c1737fba
2	Ren, 2025	https://firebasestorage.googleapis.com/v0/b/xpert-opinion-3487b.appspot.com/o/TCA%2FBase%2FRen_2025.pdf?alt=media&token=02e1bd3e-cae2-4f0c-a882-7b159e7f6319
2	Teixeira, 2023	https://firebasestorage.googleapis.com/v0/b/xpert-opinion-3487b.appspot.com/o/TCA%2FBase%2FTeixeira_2023.pdf?alt=media&token=0c9cceff-9ba7-48ba-b954-edce0d8b7f46
3	Alanis, 2024	https://firebasestorage.googleapis.com/v0/b/xpert-opinion-3487b.appspot.com/o/TCA%2FBase%2FAlanis_2024.pdf?alt=media&token=f0cda3d6-dd1f-482e-b9ec-1292b244aa63
3	Green, 2020	https://firebasestorage.googleapis.com/v0/b/xpert-opinion-3487b.appspot.com/o/TCA%2FBase%2FGreen_2020.pdf?alt=media&token=734b809a-735c-4865-9677-0081dd3c5a3b
3	Griffen, 2023	https://firebasestorage.googleapis.com/v0/b/xpert-opinion-3487b.appspot.com/o/TCA%2FBase%2FGriffen_2023.pdf?alt=media&token=6e147722-7199-40d1-a319-0396e9242e41
3	Lopes, 2024	https://firebasestorage.googleapis.com/v0/b/xpert-opinion-3487b.appspot.com/o/TCA%2FBase%2FLopes_2024.pdf?alt=media&token=2d066897-93b4-43d2-97cd-f6aa42fc1932

3	Martin, 2017	https://firebasestorage.googleapis.com/v0/b/xpert-opinion-3487b.appspot.com/o/TCA%2FBase%2FMartin_2017.pdf?alt=media&token=1780b55d-0b82-48ac-8953-29e0f889d0cf
3	McDonald, 2018	https://firebasestorage.googleapis.com/v0/b/xpert-opinion-3487b.appspot.com/o/TCA%2FBase%2FMcDonald_2018.pdf?alt=media&token=d59c991a-e3be-4d58-a024-d5e14bf2677a
4	Cañete, 2020	https://firebasestorage.googleapis.com/v0/b/xpert-opinion-3487b.appspot.com/o/TCA%2FParticipants%2F67f45fe83e26a1ee2ad84355%2FCa%C3%B1ete%2C%202020?alt=media&token=fe1242e2-1c16-4106-a7a6-3c2762671a4e
4	Kaul, 2020	https://firebasestorage.googleapis.com/v0/b/xpert-opinion-3487b.appspot.com/o/TCA%2FBase%2FKaul_2020.pdf?alt=media&token=89540e82-35af-47a8-a79f-e8231a06979d
4	Kaul, 2020a	https://firebasestorage.googleapis.com/v0/b/xpert-opinion-3487b.appspot.com/o/TCA%2FParticipants%2F67f45faeb2a6cb8f75975e7d%2FKaul%2C%202020a?alt=media&token=6f2e5432-abca-4b65-b4c3-7a9a86096a9c
4	Kverneland, 2019	https://firebasestorage.googleapis.com/v0/b/xpert-opinion-3487b.appspot.com/o/TCA%2FBase%2FKverneland_2019.pdf?alt=media&token=e0c1162a-fff3-4055-aed0-46edaaae9366
4	Lin, 2017	https://firebasestorage.googleapis.com/v0/b/xpert-opinion-3487b.appspot.com/o/TCA%2FBase%2FLin_2017.pdf?alt=media&token=4abbea17-7860-4797-8e6e-fd713885fcf5
5	Cañete, 2020a	https://firebasestorage.googleapis.com/v0/b/xpert-opinion-3487b.appspot.com/o/TCA%2FParticipants%2F67f45fe83e26a1ee2ad84355%2FCa%C3%B1ete%2C%202020a?alt=media&token=1a411bb4-0dcf-4af9-b070-f501bc9e046b
5	Kaul, 2020c	https://firebasestorage.googleapis.com/v0/b/xpert-opinion-3487b.appspot.com/o/TCA%2FParticipants%2F67f45ff82db61fdea717c6df%2FKaul%2C%202020c?alt=media&token=61a44789-8158-487c-8a24-f2c34c77c94d
5	Kverneland, 2019	https://firebasestorage.googleapis.com/v0/b/xpert-opinion-3487b.appspot.com/o/TCA%2FBase%2FKverneland_2019.pdf?alt=media&token=e0c1162a-fff3-4055-aed0-46edaaae9366
5	Lin, 2017	https://firebasestorage.googleapis.com/v0/b/xpert-opinion-3487b.appspot.com/o/TCA%2FBase%2FLin_2017.pdf?alt=media&token=4abbea17-7860-4797-8e6e-fd713885fcf5
5	Magnhild, 2019	https://firebasestorage.googleapis.com/v0/b/xpert-opinion-3487b.appspot.com/o/TCA%2FParticipants%2F67f45fe83e26a1ee2ad84355%2FMagnhild%2C%202019?alt=media&token=725c610d-824e-4e42-9d6f-5b7b4e8246fb
6	Alanis, 2024	https://firebasestorage.googleapis.com/v0/b/xpert-opinion-3487b.appspot.com/o/TCA%2FBase%2FAlanis_2024.pdf?alt=media&token=f0cda3d6-dd1f-482e-b9ec-1292b244aa63
6	Armero, 2014	https://firebasestorage.googleapis.com/v0/b/xpert-opinion-3487b.appspot.com/o/TCA%2FParticipants%2F67f45fe83e26a1ee2ad84355%2FArmero%2C%202014?alt=media&token=02bbe916-0c5a-4fb8-9d16-97de201e9ccb
6	Bara, 2023	https://firebasestorage.googleapis.com/v0/b/xpert-opinion-3487b.appspot.com/o/TCA%2FBase%2FBara_2023.pdf?alt=media&token=f4b07523-3566-49a6-9bd6-9619e798df87
6	Cervenka, 2016	https://firebasestorage.googleapis.com/v0/b/xpert-opinion-3487b.appspot.com/o/TCA%2FBase%2FCervenka_2016.pdf?alt=media&token=d6b49338-41a4-49b2-bedb-20b7fe97f200

6	Kossoff, 2015	https://firebasestorage.googleapis.com/v0/b/xpert-opinion-3487b.appspot.com/o/TCA%2FParticipants%2F67f45ff82db61fdea717c6df%2FKossoff%2C%202015?alt=media&token=32a90e64-6d35-49a4-a181-6a563db08e7b
6	Lambert, 2021	https://firebasestorage.googleapis.com/v0/b/xpert-opinion-3487b.appspot.com/o/TCA%2FBase%2FLambert_2021.pdf?alt=media&token=7482750b-2c86-49cd-b075-7577a83eb2af
6	Lima, 2020	https://firebasestorage.googleapis.com/v0/b/xpert-opinion-3487b.appspot.com/o/TCA%2FBase%2FLima_2020.pdf?alt=media&token=07c09d40-06c1-4fc7-9fee-a2add3571009
6	Schoeler, 2024	https://firebasestorage.googleapis.com/v0/b/xpert-opinion-3487b.appspot.com/o/TCA%2FBase%2FSchoeler_2024.pdf?alt=media&token=ecf8f866-3515-43ff-9338-1e2e5386f2f2
7	Gloss, 2021	https://firebasestorage.googleapis.com/v0/b/xpert-opinion-3487b.appspot.com/o/TCA%2FBase%2FGloss_2021.pdf?alt=media&token=c7f4f50e-2dfa-4b70-9999-c882c51b7b8a
7	Italian LAE, 2013	https://firebasestorage.googleapis.com/v0/b/xpert-opinion-3487b.appspot.com/o/TCA%2FParticipants%2F67f45faeb2a6cb8f75975e7d%2FItalian%20LAE%2C%202013?alt=media&token=130dd806-3b26-4ade-9b08-82e3090d48b5
7	Lallana, 2024	https://firebasestorage.googleapis.com/v0/b/xpert-opinion-3487b.appspot.com/o/TCA%2FBase%2FLallana_2024.pdf?alt=media&token=697090d0-84c2-40e0-9953-6b8da43510d0
7	Laue, 2021	https://firebasestorage.googleapis.com/v0/b/xpert-opinion-3487b.appspot.com/o/TCA%2FBase%2FLaue_2021.pdf?alt=media&token=08ec2713-b076-44a0-a1ac-bf18dbc1b49d
7	Schmidt, 2017	https://firebasestorage.googleapis.com/v0/b/xpert-opinion-3487b.appspot.com/o/TCA%2FBase%2FSchmidt_2017.pdf?alt=media&token=80662173-749b-4343-88c2-72f3999c13f8
7	Zhang, 2018	https://firebasestorage.googleapis.com/v0/b/xpert-opinion-3487b.appspot.com/o/TCA%2FBase%2FZhang_2018.pdf?alt=media&token=2cbb94ff-d923-484f-96e1-da8d417d3c93
7	ferreira Atuesta, 2023	https://firebasestorage.googleapis.com/v0/b/xpert-opinion-3487b.appspot.com/o/TCA%2FParticipants%2F67f45fc0c4c0f9c6f5e41ee4%2Fferreira%20Atuesta%2C%202023?alt=media&token=5b5626b1-4912-4bb1-8cd7-976f49d11cf9
8	Abassi, 2025	https://firebasestorage.googleapis.com/v0/b/xpert-opinion-3487b.appspot.com/o/TCA%2FBase%2FAbassi_2025.pdf?alt=media&token=d43b3a53-b386-4910-bbf8-903da6a116c4
8	Armeno, 2023	https://firebasestorage.googleapis.com/v0/b/xpert-opinion-3487b.appspot.com/o/TCA%2FBase%2FArmeno_2023.pdf?alt=media&token=e7584c47-750d-46ab-bab4-be2561149cea
8	Christensen, 2020	https://firebasestorage.googleapis.com/v0/b/xpert-opinion-3487b.appspot.com/o/TCA%2FBase%2FChristensen_2020.pdf?alt=media&token=d457ade1-03f4-446e-8426-b6232a12239e
8	El-Shafie, 2023a	https://firebasestorage.googleapis.com/v0/b/xpert-opinion-3487b.appspot.com/o/TCA%2FParticipants%2F67f45f9a3e26a1ee2ad672bc%2FEl-Shafie%2C%202023a?alt=media&token=fbfef5d2-b83b-4e8d-9ac0-09064c18fb40
8	Griffen, 2024	https://firebasestorage.googleapis.com/v0/b/xpert-opinion-3487b.appspot.com/o/TCA%2FParticipants%2F67f45f9a3e26a1ee2ad672bc%2FGriffen%2C%202024?alt=media&token=6b2648ba-a0d4-4cb5-b7ce-b1e634262bc2

8	Li, 2024	https://firebasestorage.googleapis.com/v0/b/xpert-opinion-3487b.appspot.com/o/TCA%2FBase%2FLi_2024.pdf?alt=media&token=585e0d1d-f0fb-4107-bc94-ac6771337578
8	Li, 2024a	https://firebasestorage.googleapis.com/v0/b/xpert-opinion-3487b.appspot.com/o/TCA%2FParticipants%2F67f45faeb2a6cb8f75975e7d%2FLi%2C%202024a?alt=media&token=c354ce06-b13a-4ba9-abff-6b71d16e7e07
8	Mala Manral, 2023	https://firebasestorage.googleapis.com/v0/b/xpert-opinion-3487b.appspot.com/o/TCA%2FParticipants%2F67f45fe83e26a1ee2ad84355%2FMala%20Manral%2C%202023?alt=media&token=dda8e4ee-dc3a-480d-8aed-6d772d893ae6
8	Martin, 2016	https://firebasestorage.googleapis.com/v0/b/xpert-opinion-3487b.appspot.com/o/TCA%2FBase%2FMartin_2016.pdf?alt=media&token=18bc3c90-a101-454b-b7db-3fa035f6e560
8	Martin, 2018	https://firebasestorage.googleapis.com/v0/b/xpert-opinion-3487b.appspot.com/o/TCA%2FBase%2FMartin_2018.pdf?alt=media&token=1e189d36-50bd-4954-8bd9-76040d6a5047
8	Martin, 2020	https://firebasestorage.googleapis.com/v0/b/xpert-opinion-3487b.appspot.com/o/TCA%2FBase%2FMartin_2020.pdf?alt=media&token=7dca3dd2-72d9-40db-945b-838e61e0be84
8	Mutarelli, 2023	https://firebasestorage.googleapis.com/v0/b/xpert-opinion-3487b.appspot.com/o/TCA%2FBase%2FMutarelli_2023.pdf?alt=media&token=98a17caf-cff5-4428-87d2-fab2032e3422
8	Rezaeil, 2018	https://firebasestorage.googleapis.com/v0/b/xpert-opinion-3487b.appspot.com/o/TCA%2FBase%2FRezaeil_2018.pdf?alt=media&token=8e769ac9-f177-4e71-a5b8-c080f479d929
8	Roehl, 2019	https://firebasestorage.googleapis.com/v0/b/xpert-opinion-3487b.appspot.com/o/TCA%2FParticipants%2F67f4600ac4c0f9c6f5e62e66%2FRoehl%2C%202019?alt=media&token=57969e7a-846a-43df-b1b9-94d1c5e5fa95
9	Abassi, 2025	https://firebasestorage.googleapis.com/v0/b/xpert-opinion-3487b.appspot.com/o/TCA%2FBase%2FAbassi_2025.pdf?alt=media&token=d43b3a53-b386-4910-bbf8-903da6a116c4
9	Armeno, 2023	https://firebasestorage.googleapis.com/v0/b/xpert-opinion-3487b.appspot.com/o/TCA%2FBase%2FArmeno_2023.pdf?alt=media&token=e7584c47-750d-46ab-bab4-be2561149cea
9	El-Shafie, 2023	https://firebasestorage.googleapis.com/v0/b/xpert-opinion-3487b.appspot.com/o/TCA%2FParticipants%2F67f45f9a3e26a1ee2ad672bc%2FEl-Shafie%2C%202023?alt=media&token=aa3f8a55-3b72-4282-9e62-1ff6e3d30447
9	Griffen, 2024a	https://firebasestorage.googleapis.com/v0/b/xpert-opinion-3487b.appspot.com/o/TCA%2FParticipants%2F67f45f9a3e26a1ee2ad672bc%2FGriffen%2C%202024a?alt=media&token=a7d99c1e-bdc9-4d90-8338-7dd322658da5
9	Lado, 2024	https://firebasestorage.googleapis.com/v0/b/xpert-opinion-3487b.appspot.com/o/TCA%2FBase%2FLado_2024.pdf?alt=media&token=b308d178-08b0-4fe3-98e6-233946fec9d1
9	Martin, 2016	https://firebasestorage.googleapis.com/v0/b/xpert-opinion-3487b.appspot.com/o/TCA%2FBase%2FMartin_2016.pdf?alt=media&token=18bc3c90-a101-454b-b7db-3fa035f6e560
9	Martin, 2018	https://firebasestorage.googleapis.com/v0/b/xpert-opinion-3487b.appspot.com/o/TCA%2FBase%2FMartin_2018.pdf?alt=media&token=1e189d36-50bd-4954-8bd9-76040d6a5047

9	Martin, 2020	https://firebasestorage.googleapis.com/v0/b/xpert-opinion-3487b.appspot.com/o/TCA%2FBase%2FMartin_2020.pdf?alt=media&token=7dca3dd2-72d9-40db-945b-838e61e0be84
9	Mutarelli, 2023	https://firebasestorage.googleapis.com/v0/b/xpert-opinion-3487b.appspot.com/o/TCA%2FBase%2FMutarelli_2023.pdf?alt=media&token=98a17caf-cff5-4428-87d2-fab2032e3422
9	Riccardi, 2022	https://firebasestorage.googleapis.com/v0/b/xpert-opinion-3487b.appspot.com/o/TCA%2FParticipants%2F67f4600ac4c0f9c6f5e62e66%2FRiccardi%2C%202022?alt=media&token=38f3d024-f7c3-4e45-8483-fe6d49fc581b
9	Silva, 2023	https://firebasestorage.googleapis.com/v0/b/xpert-opinion-3487b.appspot.com/o/TCA%2FParticipants%2F67f45fe83e26a1ee2ad84355%2FSilva%2C%202023?alt=media&token=086be050-d625-4bef-9774-28b7a69580d8
9	Tanya , 2023	https://firebasestorage.googleapis.com/v0/b/xpert-opinion-3487b.appspot.com/o/TCA%2FParticipants%2F67f45fe83e26a1ee2ad84355%2FTanya%20%2C%202023?alt=media&token=d145c4cb-3dcd-4250-9c47-30a4d68b74f4
10	Cervenka, 2016	https://firebasestorage.googleapis.com/v0/b/xpert-opinion-3487b.appspot.com/o/TCA%2FBase%2FCervenka_2016.pdf?alt=media&token=d6b49338-41a4-49b2-bedb-20b7fe97f200
10	Cervenka, 2016a	https://firebasestorage.googleapis.com/v0/b/xpert-opinion-3487b.appspot.com/o/TCA%2FParticipants%2F67f45faeb2a6cb8f75975e7d%2FCervenka%2C%202016a?alt=media&token=1de10bdc-6d48-459c-a8f6-6786d63ee183
10	Kaul , 2020	https://firebasestorage.googleapis.com/v0/b/xpert-opinion-3487b.appspot.com/o/TCA%2FParticipants%2F67f45faeb2a6cb8f75975e7d%2FKaul%20%2C%202020?alt=media&token=550b979a-95f1-406e-a52e-97e3d6beb3f0
10	Kaul, 2020	https://firebasestorage.googleapis.com/v0/b/xpert-opinion-3487b.appspot.com/o/TCA%2FBase%2FKaul_2020.pdf?alt=media&token=89540e82-35af-47a8-a79f-e8231a06979d
10	Kossoff, 2008	https://firebasestorage.googleapis.com/v0/b/xpert-opinion-3487b.appspot.com/o/TCA%2FParticipants%2F67f45fe83e26a1ee2ad84355%2FKossoff%2C%202008?alt=media&token=35075c6e-463a-4f7e-8a76-608f560e54c5
10	Kossoff, 2018	https://firebasestorage.googleapis.com/v0/b/xpert-opinion-3487b.appspot.com/o/TCA%2FParticipants%2F67f45ff82db61fdea717c6df%2FKossoff%2C%202018?alt=media&token=74b40a6f-69b4-4d3f-b155-44fe2bc32b09
10	Martin, 2020a	https://firebasestorage.googleapis.com/v0/b/xpert-opinion-3487b.appspot.com/o/TCA%2FParticipants%2F67f45fe83e26a1ee2ad84355%2FMartin%2C%202020a?alt=media&token=e31ffd07-a5ed-4e37-8de6-87799c96bfc0
11	Alanis, 2024a	https://firebasestorage.googleapis.com/v0/b/xpert-opinion-3487b.appspot.com/o/TCA%2FParticipants%2F67f45ff82db61fdea717c6df%2FAlanis%2C%202024a?alt=media&token=129deddd-e4ad-41fe-a1b0-c27583c7400e
11	Armeno, 2023	https://firebasestorage.googleapis.com/v0/b/xpert-opinion-3487b.appspot.com/o/TCA%2FBase%2FArmeno_2023.pdf?alt=media&token=e7584c47-750d-46ab-bab4-be2561149cea
11	Cervenka, 2016	https://firebasestorage.googleapis.com/v0/b/xpert-opinion-3487b.appspot.com/o/TCA%2FBase%2FCervenka_2016.pdf?alt=media&token=d6b49338-41a4-49b2-bedb-20b7fe97f200
11	Cervenka, 2016b	https://firebasestorage.googleapis.com/v0/b/xpert-opinion-3487b.appspot.com/o/TCA%2FParticipants%2F67f45faeb2a6cb8f75975e7d%2FCervenka%2C%202016b?alt=media&token=63b16968-2e52-4d82-9672-848055ca4f9c

11	Cervenka, 2017	https://firebasestorage.googleapis.com/v0/b/xpert-opinion-3487b.appspot.com/o/TCA%2FBase%2FCervenka_2017.pdf?alt=media&token=b135e7aa-c6cd-48a4-b34d-3af7b2ea31a9
11	Cervenka, 2017a	https://firebasestorage.googleapis.com/v0/b/xpert-opinion-3487b.appspot.com/o/TCA%2FParticipants%2F67f45faeb2a6cb8f75975e7d%2FCervenka%2C%202017a?alt=media&token=a9cc4538-14ad-49d0-8d89-c1fad0939c83
11	Cuesta, 2021	https://firebasestorage.googleapis.com/v0/b/xpert-opinion-3487b.appspot.com/o/TCA%2FParticipants%2F67f45fe83e26a1ee2ad84355%2FCuesta%2C%202021?alt=media&token=3309b1ce-65e6-4c36-8807-446387d45f23
11	Kossoff, 2018a	https://firebasestorage.googleapis.com/v0/b/xpert-opinion-3487b.appspot.com/o/TCA%2FParticipants%2F67f45ff82db61fdea717c6df%2FKossoff%2C%202018a?alt=media&token=cd86c7ff-0c55-40d3-b37f-4af4cf363cea
11	Silva, 2023a	https://firebasestorage.googleapis.com/v0/b/xpert-opinion-3487b.appspot.com/o/TCA%2FParticipants%2F67f45fe83e26a1ee2ad84355%2FSilva%2C%202023a?alt=media&token=8c55d244-1238-439a-8f56-0ef30c2d35c4
12	Armero, 2023	https://firebasestorage.googleapis.com/v0/b/xpert-opinion-3487b.appspot.com/o/TCA%2FParticipants%2F67f45fe83e26a1ee2ad84355%2FArmero%2C%202023?alt=media&token=0cae9c83-8b91-4120-b415-4ecaf54b86d6
12	Cervenka, 2016	https://firebasestorage.googleapis.com/v0/b/xpert-opinion-3487b.appspot.com/o/TCA%2FBase%2FCervenka_2016.pdf?alt=media&token=d6b49338-41a4-49b2-bedb-20b7fe97f200
12	Cervenka, 2016c	https://firebasestorage.googleapis.com/v0/b/xpert-opinion-3487b.appspot.com/o/TCA%2FParticipants%2F67f45faeb2a6cb8f75975e7d%2FCervenka%2C%202016c?alt=media&token=e54dba2a-057e-4e9c-b12d-87781fe839d2
12	Cervenka, 2016d	https://firebasestorage.googleapis.com/v0/b/xpert-opinion-3487b.appspot.com/o/TCA%2FParticipants%2F67f45fe83e26a1ee2ad84355%2FCervenka%2C%202016d?alt=media&token=04e9b99e-231f-4602-b5e9-e1e4dd282d5d
12	Kossoff, 2008a	https://firebasestorage.googleapis.com/v0/b/xpert-opinion-3487b.appspot.com/o/TCA%2FParticipants%2F67f45fe83e26a1ee2ad84355%2FKossoff%2C%202008a?alt=media&token=e9812d71-ddd1-4582-8fb7-ce8074f4b16f
13	Armeno, 2023	https://firebasestorage.googleapis.com/v0/b/xpert-opinion-3487b.appspot.com/o/TCA%2FBase%2FArmeno_2023.pdf?alt=media&token=e7584c47-750d-46ab-bab4-be2561149cea
13	Cervenka, 2017	https://firebasestorage.googleapis.com/v0/b/xpert-opinion-3487b.appspot.com/o/TCA%2FBase%2FCervenka_2017.pdf?alt=media&token=b135e7aa-c6cd-48a4-b34d-3af7b2ea31a9
13	Cervenka, 2017b	https://firebasestorage.googleapis.com/v0/b/xpert-opinion-3487b.appspot.com/o/TCA%2FParticipants%2F67f45faeb2a6cb8f75975e7d%2FCervenka%2C%202017b?alt=media&token=60161cfe-ddc3-45b6-8dc9-8cc38a8d68ba
13	Kaul, 2020b	https://firebasestorage.googleapis.com/v0/b/xpert-opinion-3487b.appspot.com/o/TCA%2FParticipants%2F67f45fe83e26a1ee2ad84355%2FKaul%2C%202020b?alt=media&token=045981f7-3288-48e8-ac21-33e9a649235d
13	Kossoff, 2008b	https://firebasestorage.googleapis.com/v0/b/xpert-opinion-3487b.appspot.com/o/TCA%2FParticipants%2F67f45fe83e26a1ee2ad84355%2FKossoff%2C%202008b?alt=media&token=d99e4ac5-aca5-40c1-9735-6fc83d5cfa1e

https://doi.org/10.3390/nu18203458

Supplementary S2. Complete Search Strategies for Each Clinical Question and Database

Question 1

In adult patients with drug-resistant epilepsy in whom a clinically significant reduction is sought, what are the relevant clinical characteristics for prescribing ketogenic therapy with a commercial formula?

Pubmed

("Drug-resistant epilepsy"[Mesh] OR "Drug-resistant epilepsy"[Title/Abstract] OR "Drug Resistant Epilepsies"[Title/Abstract] OR "Intractable Epilepsy"[Title/Abstract] OR "Refractory Epilepsies"[Title/Abstract] OR "Drug Refractory Epilepsy"[Title/Abstract] OR "Medication Resistant Epilepsy"[Title/Abstract]) AND ("Ketocal*"[Title/Abstract] OR "Liquigen*"[Title/Abstract] OR "Diet, Ketogenic"[Mesh] OR "Ketogenic Diet"[Title/Abstract] OR "Ketogenic Diets"[Title/Abstract]) AND (("Seizures"[Mesh] OR "Seizures"[Title/Abstract]) AND ("prevention and control"[Title/Abstract] OR "Treatment Outcome"[Mesh] OR "Treatment Outcome"[Title/Abstract] OR "Therapeutics"[Mesh] OR "Secondary Prevention"[Mesh] OR "Tertiary Prevention"[Mesh]))

Embase

('refractory epilepsy'/mj OR 'drug refractory epilepsy':ab,ti OR 'drug-resistant epilepsy':ab,ti OR 'intractable epilepsy':ab,ti OR 'pharmaco-refractory epilepsy':ab,ti OR 'treatment resistant epilepsy':ab,ti) AND ('Functional Formularies Keto':ab,ti OR 'Ketocal 4:1':ab,ti OR 'Ketocal':ab,ti OR 'KetoVOLVE':ab,ti OR 'Functional Formularies Keto®':ab,ti OR 'Ketocal®':ab,ti OR 'KetoVOLVE®':ab,ti OR 'KetoCal 3:1':ab,ti OR 'Liquigen':ab,ti OR 'K·Quik™':ab,ti OR 'KetoVie':ab,ti OR 'ketogenic diet'/mj) AND ('seizure control'/exp/mj OR 'seizure'/mj OR 'seizures':ab,ti OR 'seizure':ab,ti) AND ('disease control'/mj OR 'secondary prevention'/mj OR 'therapy'/mj OR 'treatment outcome'/mj OR 'treatment outcome':ab,ti) AND ([adult]/lim OR [young adult]/lim OR [middle aged]/lim OR [aged]/lim OR [very elderly]/lim) AND [2015-2025]/py

Question 2

https://doi.org/10.3390/nu18203458

In adult patients with super-refractory status epilepticus (SRSE) or cluster seizures in whom seizure control with ketogenic therapy is sought, what are the indications for prescribing this therapy with a commercial formula?

Pubmed

("Status
Epilepticus"[Mesh] OR "Generalized Convulsive Status
Epilepticus"[Title/Abstract] OR "Super-refractory status
epilepticus"[Title/Abstract] OR "Super refractory status
epilepticus"[Title/Abstract] OR "SRSR"[Title/Abstract] OR
"Cluster seizures"[Title/Abstract] OR "Clustered
seizures"[Title/Abstract]) AND ("Ketocal*"[Title/Abstract] OR
"Liquigen*"[Title/Abstract] OR "Diet, Ketogenic"[Mesh] OR
"Ketogenic Diet"[Title/Abstract] OR "Ketogeni
Diets"[Title/Abstract]) AND (("Seizures"[Mesh] OR"Seizures"[Title/Abstract])
AND ("prevention and control"[Title/Abstract] OR "Treatment
Outcome"[Mesh] OR "Treatment Outcome"[Title/Abstract] OR
"Therapeutics"[Mesh] OR "Secondary Prevention"[Mesh] OR
"Tertiary Prevention"[Mesh]))

Embase

('convulsive status
epilepticus'/mj OR 'Generalized Convulsive Status Epilepticus':ab,ti OR
'refractory status epilepticus'/mj OR 'refractory epilepsy'/mj OR 'super
refractory status epilepticus'/mj OR 'super refractory status
epilepticus':ab,ti OR 'refractory status epilepticus':ab,ti OR 'Cluster
seizures':ab,ti OR 'Clustered seizures':ab,ti) AND ('Liquigen':ab,ti OR
'KetoVie':ab,ti OR 'ketogenic diet'/mj OR 'ketogenic diet':ab,ti) AND ('seizure
control'/exp/mj OR 'seizure'/mj OR 'seizures':ab,ti OR 'seizure':ab,ti) AND
([adult]/lim OR [young adult]/lim OR [middle aged]/lim OR [aged]/lim OR [very
elderly]/lim) AND [2015-2025]/py

Question 3

In adult patients with drug-resistant epilepsy who are candidates for ketogenic therapy, what elements should be included in the ketogenic therapy initiation protocol to achieve an adequate level of adherence?

Pubmed

("Ketocal*"[Title/Abstract]
OR "Liquigen*"[Title/Abstract] OR "Diet, Ketogenic"[Mesh]
OR "Ketogenic Diet"[Title/Abstract] OR "Ketogenic

https://doi.org/10.3390/nu18203458

Diets"[Title/Abstract]) AND ("Assessment of Medication Adherence"[Mesh] OR "Guideline Adherence"[Mesh] OR "Adherence Interventions"[Mesh] OR "Treatment Adherence and Compliance"[Mesh] OR "Medication Adherence"[Mesh] OR "Prescription Adherence"[Title/Abstract] OR "Adherence Interventions"[Title/Abstract] OR "Adherence Support Interventions"[Title/Abstract])

Embase

('refractory epilepsy'/mj
OR 'drug refractory epilepsy':ab,ti OR 'drug-resistant epilepsy':ab,ti OR 'intractable epilepsy':ab,ti OR 'pharmaco-refractory epilepsy':ab,ti OR 'treatment resistant epilepsy':ab,ti) AND ('Liquigen':ab,ti OR 'KetoVie':ab,ti OR 'ketogenic diet'/exp/mj OR 'ketogenic diet':ab,ti) AND ('patient compliance'/exp/mj OR 'adherence to therapy':ab,ti OR 'adherence to treatment':ab,ti OR 'patient adherence':ab,ti) AND ([adult]/lim OR [young adult]/lim OR [middle aged]/lim OR [aged]/lim OR [very elderly]/lim) AND [2015-2025]/py

Question 4

In hospitalized adult patients (emergency department, ward, or ICU) with drug-resistant epilepsy receiving ketogenic therapy, which medications, when coadministered, are considered high risk for causing severe interactions or may prevent stable ketosis (β-hydroxybutyrate >2 mmol/L)?

Pubmed

("Drug-resistant
epilepsy"[Mesh] OR "Drug-resistant epilepsy"[Title/Abstract] OR "Drug Resistant Epilepsies"[Title/Abstract] OR "Intractable Epilepsy"[Title/Abstract] OR "Refractory Epilepsies"[Title/Abstract] OR "Drug Refractory Epilepsy"[Title/Abstract] OR "Medication Resistant Epilepsy"[Title/Abstract]) AND ("Diet, Ketogenic"[Mesh] OR "Ketogenic Diet"[Title/Abstract] OR "Ketogenic Diets"[Title/Abstract]) AND ("Drug interaction"[Title/Abstract] OR "Drug Interactions"[Mesh] OR "Drug-Related Side Effects and Adverse Reactions"[Mesh] OR "Drug Incompatibility"[Mesh] OR "adverse effects" [Subheading] OR "Adverse drug interaction"[Title/Abstract] OR "Harmful drug interaction"[Title/Abstract])

Embase

https://doi.org/10.3390/nu18203458

('refractory epilepsy'/exp/mj OR 'drug refractory epilepsy':ab,ti OR 'drug-resistant epilepsy':ab,ti OR 'intractable epilepsy':ab,ti OR 'pharmaco-refractory epilepsy':ab,ti OR 'treatment resistant epilepsy':ab,ti) AND ('ketogenic diet'/exp/mj OR 'ketogenic diet':ab,ti) AND ('drug interaction'/mj OR 'drug interaction':ab,ti OR 'adverse drug reaction'/mj OR 'adverse drug reaction':ab,ti OR 'adverse drug effect':ab,ti OR 'drug incompatibility'/mj OR 'adverse event'/mj OR 'adverse event':ab,ti) AND ([adult]/lim OR [young adult]/lim OR [middle aged]/lim OR [aged]/lim OR [very elderly]/lim) AND [2015-2025]/py

Question 5

In adult outpatients with drug-resistant epilepsy receiving ketogenic therapy, which medications, when coadministered, are considered high risk for causing severe interactions or may prevent stable ketosis (β-hydroxybutyrate >2 mmol/L)?

Pubmed

("Diet, Ketogenic"[Mesh] OR "Ketogenic Diet"[Title/Abstract] OR "Ketogenic Diets"[Title/Abstract] OR “Modified Atkins Diet”[Title/Abstract]) AND ("Drug interaction"[Title/Abstract] OR "Drug Interactions"[Mesh] OR "Drug-Related Side Effects and Adverse Reactions"[Mesh] OR "Drug Incompatibility"[Mesh] OR "adverse effects" [Subheading] OR "Adverse drug interaction"[Title/Abstract] OR "Harmful drug interaction"[Title/Abstract])

Embase

('refractory epilepsy'/exp/mj OR 'drug refractory epilepsy':ab,ti OR 'drug-resistant epilepsy':ab,ti OR 'intractable epilepsy':ab,ti OR 'pharmaco-refractory epilepsy':ab,ti OR 'treatment resistant epilepsy':ab,ti) AND ('ketogenic diet'/exp/mj OR 'ketogenic diet':ab,ti) AND ('drug interaction'/mj OR 'drug interaction':ab,ti OR 'adverse drug reaction'/mj OR 'adverse drug reaction':ab,ti OR 'adversedrug effect':ab,ti OR 'drug incompatibility'/mj OR 'adverse event'/mj OR 'adverse event':ab,ti) AND ([adult]/lim OR [young adult]/lim OR [middle aged]/lim OR [aged]/lim OR [very elderly]/lim) AND [2015-2025]/py

Question 6

https://doi.org/10.3390/nu18203458

In adult patients with drug-resistant epilepsy receiving ketogenic therapy, which healthcare professionals should comprise the structured follow-up team to improve treatment adherence, seizure control, and management of adverse effects?

Pubmed

(("Drug-resistant
epilepsy"[Mesh] OR "Drug-resistant epilepsy"[Title/Abstract] OR
"Drug Resistant

Epilepsies"[Title/Abstract]
OR "Intractable Epilepsy"[Title/Abstract] OR "Refractory
Epilepsies"[Title/Abstract] OR "Drug Refractory
Epilepsy"[Title/Abstract] OR "Medication Resistant

Epilepsy"[Title/Abstract])
AND ("Diet, Ketogenic"[Mesh] OR "Ketogenic
Diet"[Title/Abstract] OR "Ketogenic Diets"[Title/Abstract])) AND
("Case Management"[Mesh] OR "Secondary Care"[Mesh] OR
"Managed Care Programs"[Mesh] OR "Patient Care
Planning"[Mesh] OR "Follow up"[Title/Abstract])

Embase

('refractory epilepsy'/mj
OR 'drug refractory epilepsy':ab,ti OR 'drug-resistant epilepsy':ab,ti OR
'intractable epilepsy':ab,ti OR 'pharmaco-refractory epilepsy':ab,ti OR
'treatment resistant epilepsy':ab,ti) AND ('ketogenic diet'/mj OR 'ketogenic
diet':ab,ti) AND ('follow up'/mj OR 'case management'/mj OR 'follow up':ab,ti
OR 'follow up team':ab,ti OR 'patient care planning'/mj OR 'patient care
planning':ab,ti) AND ([adult]/lim OR [young adult]/lim OR [middle aged]/lim OR
[aged]/lim OR [very elderly]/lim) AND [2015-2025]/py

Question 7

In adult patients with drug-resistant epilepsy who have been seizure-free for ≥2 years while receiving MAC treatment, what considerations should be taken into account when gradually reducing the MAC dose to maintain seizure control and reduce the risk of recurrence?

Pubmed

("Drug-resistant
epilepsy"[Mesh] OR "Drug-resistant epilepsy"[Title/Abstract] OR
"Drug Resistant Epilepsies"[Title/Abstract] OR "Intractable
Epilepsy"[Title/Abstract] OR "Refractory Epilepsies"[Title/Abstract]

OR "Drug Refractory Epilepsy"[Title/Abstract] OR "Medication Resistant Epilepsy"[Title/Abstract]) AND ("Anticonvulsants"[Mesh] OR "Epilepsy/drug therapy"[Mesh] OR "Antiepileptic drug"[Title/Abstract] OR "Anticonvulsants"[Title/Abstract]) AND ("Drug Withdrawal"[Title/Abstract] OR "Withdrawal"[Title/Abstract] OR "Tapering"[Title/Abstract] OR "Discontinuation"[Title/Abstract] OR "Dose reduction"[Title/Abstract] OR "Anticonvulsant withdrawal"[Title/Abstract] OR "Antiepileptic drug tapering"[Title/Abstract])

Embase

('refractory epilepsy'/mj
OR 'drug refractory epilepsy':ab,ti OR 'drug-resistant epilepsy':ab,ti OR 'intractable epilepsy':ab,ti OR 'pharmaco-refractory epilepsy':ab,ti OR 'treatment resistant epilepsy':ab,ti) AND ('seizure freedom'/mj OR 'Seizure remission':ab,ti OR '2-year remission':ab,ti OR '24-month remission':ab,ti) AND ('recurrence risk'/mj OR 'recurrence risk':ab,ti OR 'relapse'/mj OR 'relapse':ab,ti OR 'seizure recurrence':ab,ti OR 'seizure relapse':ab,ti OR 'seizure contro':ab,ti OR

'therapy effect'/mj) AND
([adult]/lim OR [young adult]/lim OR [middle aged]/lim OR [aged]/lim OR [very elderly]/lim) AND [2015-2025]/py

Question 8

In adult patients with drug-resistant epilepsy for whom classical ketogenic therapy is not the most advisable option, which of the modified ketogenic therapies (modified Atkins diet, medium-chain triglyceride diet, and low-glycemic-index treatment) is the most recommended?

Pubmed

("Drug-resistant epilepsy"[Mesh]OR"Drug Refractory Epilepsy"[All fields]OR"Refractory Epilepsies"[All fields]OR"Intractable Epilepsies"[All fields]OR"Medication Resistant Epilepsy"[All fields]OR"Refractory Epilepsy"[All fields]OR"Resistant Epilepsy"[All fields]OR"Epilepsy"[All fields]OR"Epilepsies"[All fields]OR"Seizure Disorder"[All fields]) AND ("Diet, Ketogenic"[Mesh]OR"Ketogenic Diet"[All fields]OR"Diets, Ketogenic"[All fields]OR"Ketogenic Diets"[All fields])

Embase

("Drug-resistant epilepsy"OR"Drug Refractory Epilepsy"OR"Refractory Epilepsies"OR"Intractable Epilepsies"OR"Medication Resistant Epilepsy"OR"Refractory Epilepsy"OR"Resistant Epilepsy"OR"Epilepsy"OR"Epilepsies"OR"Seizure Disorder") AND ("Diet, Ketogenic""Ketogenic Diet""Diets, Ketogenic""Ketogenic Diets")

Question 9

In patients with drug-resistant epilepsy and high cardiovascular risk receiving ketogenic therapy, what would be the most appropriate profile of dietary fats to promote a reduction in LDL cholesterol?

Pubmed

("Drug Refractory Epilepsy"[All fields]OR"Refractory Epilepsies"[All fields]OR"Intractable Epilepsies"[All fields]OR"Medication Resistant Epilepsy"[All fields]OR"Refractory Epilepsy"[All fields]OR"Resistant Epilepsy"[All fields]OR"Epilepsy"[All fields]OR"Epilepsies"[All fields]OR"Seizure Disorder"[All fields]) AND ("Cardiovascular Risk"[All fields]) AND ("Diet, Ketogenic"[Mesh]OR"Ketogenic Diet"[All fields]OR"Diets, Ketogenic"[All fields]OR"Ketogenic Diets"[All fields])

Embase

("Drug Refractory Epilepsy"OR"Refractory Epilepsies"OR"Intractable Epilepsies"OR"Medication Resistant Epilepsy"OR"Refractory Epilepsy"OR"Resistant Epilepsy"OR"Epilepsy"OR"Epilepsies"OR"Seizure Disorder") AND ("Ketogenic Diet"OR"Diets, Ketogenic"OR"Ketogenic Diets""Cardiovascular Risk")

Question 10

In adult patients with drug-resistant epilepsy treated with ketogenic therapy, what type of vitamin and mineral supplementation should they receive to prevent nutritional deficiencies and optimize metabolic status?

Pubmed

https://doi.org/10.3390/nu18203458

("Drug-resistant epilepsy"[Mesh]OR"Drug Refractory Epilepsy"[All fields]OR"Refractory Epilepsies"[All fields]OR"Intractable Epilepsies"[All fields]OR"Medication Resistant Epilepsy"[All fields]OR"Refractory Epilepsy"[All fields]OR"Resistant Epilepsy"[All fields]OR"Epilepsy"[All fields]OR"Epilepsies"[All fields]OR"Seizure Disorder"[All fields]) AND ("Dietary Supplement"[All fields]OR"Nutritional Supplement"[All fields]OR"vitamin"[All fields]) AND ("Diet, Ketogenic"[Mesh]OR"Ketogenic Diet"[All fields]OR"Diets, Ketogenic"[All fields]OR"Ketogenic Diets"[All fields])

Embase

("Drug-resistant epilepsy"OR"Drug Refractory Epilepsy"OR"Refractory Epilepsies"OR"Intractable Epilepsies"OR"Medication Resistant Epilepsy"OR"Refractory Epilepsy"OR"Resistant Epilepsy"OR"Epilepsy"OR"Epilepsies"OR"Seizure Disorder") AND ("Diet, Ketogenic"OR"Ketogenic Diet"OR"Diets, Ketogenic"OR"Ketogenic Diets""Dietary Supplement"OR)

Question 11

In adult patients with drug-resistant epilepsy receiving ketogenic therapy, which laboratory tests should be included in periodic monitoring for the early detection of complications (dyslipidemia, hypocalcemia, metabolic acidosis) and the maintenance of optimal ketosis (β-hydroxybutyrate >2 mmol/L)?

Pubmed

("Drug-resistant epilepsy"[Mesh]OR"Drug Refractory Epilepsy"[All fields]OR"Refractory Epilepsies"[All fields]OR"Intractable Epilepsies"[All fields]OR"Medication Resistant Epilepsy"[All fields]OR"Refractory Epilepsy"[All fields]OR"Resistant Epilepsy"[All fields]OR"Epilepsy"[All fields]OR"Epilepsies"[All fields]OR"Seizure Disorder"[All fields]) AND ("Diet, Ketogenic"[Mesh]OR"Ketogenic Diet"[All fields]OR"Diets, Ketogenic"[All fields]OR"Ketogenic Diets"[All fields])

Embase

("Drug-resistant epilepsy"OR"Drug Refractory Epilepsy"OR"Refractory Epilepsies"OR"Intractable

https://doi.org/10.3390/nu18203458

Epilepsies"OR"Medication Resistant Epilepsy"OR"Refractory Epilepsy"OR"Resistant Epilepsy"OR"Epilepsy"OR"Epilepsies"OR"Seizure Disorder") AND ("Diet, Ketogenic"OR"Ketogenic Diet"OR"Diets, Ketogenic"OR"Ketogenic Diets")

Question 12

In patients with drug-resistant epilepsy treated with ketogenic therapy, how frequently should laboratory and nutritional assessments be performed to improve seizure control, prevent adverse effects, and optimize nutritional status?

Pubmed

("Drug-resistant epilepsy"[Mesh]OR"Drug Refractory Epilepsy"[All fields]OR"Refractory Epilepsies"[All fields]OR"Intractable Epilepsies"[All fields]OR"Medication Resistant Epilepsy"[All fields]OR"Refractory Epilepsy"[All fields]OR"Resistant Epilepsy"[All fields]OR"Epilepsy"[All fields]OR"Epilepsies"[All fields]OR"Seizure Disorder"[All fields]) AND ("Monitoring"[All fields]OR"Follow up"[All fields]) AND ("Diet, Ketogenic"[Mesh]OR"Ketogenic Diet"[All fields]OR"Diets, Ketogenic"[All fields]OR"Ketogenic Diets"[All fields])

Embase

("Drug-resistant epilepsy"OR"Drug Refractory Epilepsy"OR"Refractory Epilepsies"OR"Intractable Epilepsies"OR"Medication Resistant Epilepsy"OR"Refractory Epilepsy"OR"Resistant Epilepsy"OR"Epilepsy"OR"Epilepsies"OR"Seizure Disorder") AND ("Diet, Ketogenic"OR"Ketogenic Diet"OR"Diets, Ketogenic"OR"Ketogenic Diets")

Question 13

In patients with refractory status epilepticus for whom ketogenic therapy has been indicated, which laboratory tests should be performed to detect or prevent complications?

Pubmed

("Drug-resistant epilepsy"[Mesh]OR"Drug Refractory Epilepsy"[All fields]OR"Refractory Epilepsies"[All fields]OR"Intractable Epilepsies"[All fields]OR"Medication Resistant Epilepsy"[All fields]OR"Refractory Epilepsy"[All fields]OR"Resistant Epilepsy"[All fields]OR"Epilepsy"[All

https://doi.org/10.3390/nu18203458

fields]OR"Epilepsies"[All fields]OR"Seizure Disorder"[All fields]) AND ("Monitoring"[All fields]OR"Follow up"[All fields]) AND ("Diet, Ketogenic"[Mesh]OR"Ketogenic Diet"[All fields]OR"Diets, Ketogenic"[All fields]OR"Ketogenic Diets"[All fields]) AND ("Monitoring"[All fields]OR"Follow up"[All fields])

Embase

("Drug Refractory Epilepsy"OR"Refractory Epilepsies"OR"Intractable Epilepsies"OR"Medication Resistant Epilepsy"OR"Refractory Epilepsy"OR"Resistant Epilepsy"OR"Epilepsy"OR"Epilepsies"OR"Seizure Disorder") AND ("Ketogenic Diets""Monitoring"OR"Follow up") AND ("Diet, Ketogenic"OR"Ketogenic Diet"OR)

Bibliography.

1. Manera K, Hanson CS, Gutman T, Tong A. Consensus methods: Nominal group technique. Handbook of Research Methods in Health Social Sciences. 2019 Jan 12;737–50. doi:10.1007/978-981-10-5251-4_100/TABLES/1
2. McMillan SS, King M, Tully MP. How to use the nominal group and Delphi techniques. Int J Clin Pharm. 2016 Jun 1;38(3):655–62. doi:10.1007/S11096-016-0257-X PubMed PMID: 26846316.
3. Delbecq A, Ven AH Van de, Gustafson D. Group Techniques for Program Planning: A Guide to Nominal Group and Delphi Processes. J Appl Behav Sci. 1976 Oct;12(4):581–581. doi:10.1177/002188637601200414
4. National Institute for Health and Care Excellence Final Epilepsies in children, young people and adults [E] Monotherapy for generalised tonic-clonic and focal onset seizures NICE guideline NG217 FINAL Contents. 2022.
5. Lado FA, Ahrens SM, Riker E, Muh CR, Richardson RM, Gray J, et al. Guidelines for Specialized Epilepsy Centers. Neurology. 2024 Feb 27;102(4):e208087. doi:10.1212/WNL.0000000000208087 PubMed PMID: 38306606.
6. Jehi L, Jette N, Kwon CS, Josephson CB, Burneo JG, Cendes F, et al. Timing of referral to evaluate for epilepsy surgery: Expert Consensus Recommendations from the Surgical Therapies Commission of the International League Against Epilepsy. Epilepsia. 2022 Oct 1;63(10):2491–506. doi:10.1111/EPI.17350 PubMed PMID: 35842919.
7. De Giorgis V, Tagliabue A, Bisulli F, Brambilla I, Camerini A, Cusmai R, et al. Ketogenic dietary therapies in epilepsy: recommendations of the Italian League against Epilepsy Dietary Therapy Study Group. Front Neurol. 2023;14:1215618. doi:10.3389/FNEUR.2023.1215618/FULL
8. Cervenka MC, Wood S, Bagary M, Balabanov A, Bercovici E, Brown MG, et al. International Recommendations for the Management of Adults Treated With Ketogenic Diet Therapies. Neurol Clin Pract. 2021 Oct 1;11(5):385. doi:10.1212/CPJ.0000000000001007 PubMed PMID: 34840865.
9. De Giorgis V, Tagliabue A, Bisulli F, Brambilla I, Camerini A, Cusmai R, et al. Ketogenic dietary therapies in epilepsy: recommendations of the Italian League against Epilepsy Dietary Therapy Study Group. Front Neurol. 2023;14. doi:10.3389/FNEUR.2023.1215618 PubMed PMID: 37497012.
10. Erkent I, Ilgaz F, Dericioglu N. Difficulties in the implementation of the ketogenic diet in adult patients with refractory epilepsy. Epilepsy and Behavior. 2023 Jul 1;144. doi:10.1016/j.yebeh.2023.109234 PubMed PMID: 37192580.
11. Kossoff EH, Zupec-Kania BA, Auvin S, Ballaban-Gil KR, Christina Bergqvist AG, Blackford R, et al. Optimal clinical management of children receiving dietary therapies for epilepsy: Updated recommendations of the International Ketogenic Diet Study Group. Epilepsia Open. 2018 Jun 1;3(2):175–92. doi:10.1002/EPI4.12225 PubMed PMID: 29881797.
12. Borowicz-Reutt K, Krawczyk M, Czernia J. Ketogenic Diet in the Treatment of Epilepsy. Nutrients. 2024 Apr 24;16(9). doi:10.3390/NU16091258 PubMed PMID: 38732505.
13. Cervenka MC, Wood S, Bagary M, Balabanov A, Bercovici E, Brown MG, et al. International Recommendations for the Management of Adults Treated With Ketogenic Diet Therapies. Neurol Clin Pract. 2021 Oct 1;11(5):385–97. doi:10.1212/CPJ.0000000000001007 PubMed PMID: 34840865.
14. Ballesteros Tapias JK, Conde Hurtado DI, Castaño LH, Pérez AM. Ketogenic diet therapies as a non-pharmacological adjuvant in resistant epilepsy: retrospective analysis of adult outpatients in Colombia. Nutr Neurosci. 2024;27(12):1363–9. doi:10.1080/1028415X.2024.2336716 PubMed PMID: 38622918.

Disclaimer/Publisher's Note: The statements, opinions and data contained in all publications are solely those of the individual author(s) and contributor(s) and not of MDPI and/or the editor(s). MDPI and/or the editor(s) disclaim responsibility for any injury to people or property resulting from any ideas, methods, instructions or products referred to in the content.

https://doi.org/10.3390/nu18203458
